# Dissecting Key Adaptation Traits in the Polyploid Perennial *Medicago sativa* Using GBS-SNP Mapping

**DOI:** 10.3389/fpls.2018.00934

**Published:** 2018-07-04

**Authors:** Laxman Adhikari, Orville M. Lindstrom, Jonathan Markham, Ali M. Missaoui

**Affiliations:** ^1^Crop and Soil Sciences and Institute of Plant Breeding Genetics and Genomics, Center for Applied Genetic Technologies, University of Georgia, Athens, GA, United States; ^2^Department of Horticulture, University of Georgia, Athens, GA, United States

**Keywords:** alfalfa, genetic map, QTL, genotype X environment interaction, fall dormancy, winter hardiness

## Abstract

Understanding key adaptation traits is crucial to developing new cultivars with broad adaptations. The main objective of this research is to understand the genetic basis of winter hardiness (WH) and fall dormancy (FD) in alfalfa and the association between the two traits. QTL analysis was conducted in a pseudo-testcross F1 population developed from two cultivars contrasting in FD (3010 with *FD* = 2 and CW 1010 with *FD* = 10). The mapping population was evaluated in three replications at two locations (Watkinsville and Blairsville, GA). FD levels showed low to moderate correlations with WH (0.22–0.57). Assessing dormancy in winter is more reliable than in the fall in southern regions with warm winters. The mapping population was genotyped using Genotyping-by-sequencing (GBS). Single dose allele SNPs (SDA) were used for constructing linkage maps. The parental map (CW 1010) consisted of 32 linkage groups spanning 2127.5 cM with 1377 markers and an average marker density of 1.5 cM/SNP. The maternal map (3010) had 32 linkage groups spanning 2788.4 cM with 1837 SDA SNPs with an average marker density of 1.5 cM/SNP. Forty-five significant (*P* < 0.05) QTLs for FD and 35 QTLs for WH were detected on both male and female linkage maps. More than 75% (22/28) of the dormancy QTL detected from the 3010 parent did not share genomic regions with WH QTLs and more than 70% (12/17) dormancy QTLs detected from CW 1010 parent were localized in different genomic regions than WH QTLs. These results suggest that the two traits have independent inheritance and therefore can be improved separately in breeding programs.

## Introduction

Alfalfa (*Medicago sativa* L.) is a perennial cool-season forage legume grown worldwide for hay, pasture and silage (Duke, 1981; Adhikari and Missaoui, 2017). It is native to the southwestern and central Asia, near southern Caucasus Mountains (Duke, 1981; Li and Brummer, 2012). Alfalfa is well-regarded for providing high-quality forage with high protein content and nutritive value (Duke, 1981). Like other legumes, alfalfa fixes atmospheric nitrogen (N), up to 130–220 lbs per acre per year, thereby supplying N to itself and succeeding crops in rotation^1^. In the US., alfalfa and its mixtures contribute a major part of haylage production, where the productivity varies from 1.1 ton/acre (Rhode Island) to 7 ton/acre (California) with an average national productivity of 3.45 ton/acre in 2016^2^. Alfalfa is cross-pollinated and highly heterozygous. It is a polyploid (2n = 4x = 32) with tetrasomic inheritance and a genome size near 1 Gb (Li and Brummer, 2012). Alfalfa grows best in cool sub-tropical and warm temperate environments (Duke, 1981). Growth and yield are remarkably affected by seasonal dormancy and low temperature stress in winter (Adhikari et al., 2017).

Alfalfa evolved FD as an important adaptation strategy to survive in latitudes with harsh winter conditions. The short growth cycle of fall-dormant alfalfa varieties limits not only the amounts of biomass accumulated but also the seasonal distribution, which is reduced to a few harvests per year in summer. FD rating (FDR) of alfalfa cultivars is assigned based on fall regrowth height, after clipping, to 11 groups ranging from FD 1 to FD 11 with lower numbers indicating more dormant (Teuber et al., 1998). These groups are very dormant, (FD 1, 2); dormant (FD 3, 4), moderately dormant (FD 5), semi-dormant (FD 6, 7), non-dormant (FD 8, 9), and very non-dormant (FD 10, 11)^3^. Dormancy classes are assigned based on standard check cultivars. Diminishing day length and temperature in fall season are the two major environmental factors triggering physiological dormancy in alfalfa (McKenzie et al., 1988; Brummer et al., 2000). FD is a strongly expressed trait where certain genotypes exhibit slow growth leading to a short stature and decumbent plant architecture after autumn clipping (Teuber et al., 1998; Adhikari et al., 2017). In order to assign FD accurately in the field, it is suggested to collect information from multiple locations for least for 2 years (Teuber et al., 1998).

The genetic control of FD in alfalfa is not known and investigation into the endogenous factors influencing FD will be valuable for developing cultivars with no- or short-fall dormancy. The molecular basis of dormancy has been studied mostly in woody species adapted to temperate environments. There are few reports on QTLs associated with the dormancy trait in herbaceous forage species. Some QTLs associated with fall growth and WH were mapped using an interspecific hybrid population developed by crossing annual x perennial ryegrass (Xiong et al., 2007). Day length and temperature are most likely the two major environmental cues that plants use to sense the environmental changes (Olsen, 2010; Tanino et al., 2010). Genomic studies have identified a number of genes involved in the control of dormancy induction and growth cessation, including circadian clock regulators (Ibáñez et al., 2010). However, McKenzie et al. (1988) argued that alfalfa FD is not physiologically similar to that of higher trees since the plant exhibits dormancy due to decreasing day length and temperature but it is reversible when alfalfa is switched to an environment with warmer temperature and longer photoperiod. Research investigating the genetic and physiological basis of FD in alfalfa, in the context of genes, quantitative trait loci (QTL) and hormones regulating the process of alfalfa FD (Brouwer et al., 2000; Li and Brummer, 2012) suggested that FD in alfalfa is correlated with winter survival and very often, fall dormant alfalfa is considered more winter-hardy (Stout and Hall, 1989; Li et al., 2014a). In northern latitudes, mostly dormant germplasm is grown because they have better chances of completing the development cycle and go dormant before the onset of freezing temperatures in early fall. There is a lack of consensus regarding the relationship between fall regrowth and WH even though alfalfa breeders have been routinely using FD as a surrogate to select for cold tolerance in northern latitudes. A strong phenotypic as well as genetic correlation between dormancy and WH was observed in alfalfa breeding populations developed from wide dormancy crosses involving parents with contrasting dormancy ratings (Li et al., 2015). Cunningham et al. (1998) examined the impact of selection for differences in FD on carbohydrate and protein accumulation in roots and crown buds as well as its effect on winter survival and bud development in four alfalfa parents and their progeny. They concluded, after three cycles of selection, that imposing selection on FD will lead to improved cold acclimation and winter survival. Brummer et al. (2000) stressed the need for reexamining the relationship between FD and WH because contrary to the traditional concept, they found weak association between the two traits (Brummer et al., 2000). Similarly, quantitative trait loci (QTLs) independently controlling autumn plant growth and winter survival were reported indicating the possibility of independent improvement of the two traits through marker-assisted selection (MAS) (Li et al., 2015). In a recent study, scientists have identified differentially expressed genes such as C-repeat binding factors (CBF) in response to freezing stress in alfalfa which may be induced regardless of the genotype dormancy (Shu et al., 2017). Zhang et al. (2015) also observed several differentially expressed genes in fall dormant lines in leaf transcriptome analysis (Zhang et al., 2015). Similarly, alfalfa cold acclimation specific (*CAS*) genes such as *cas15* and other cold related genes are also potential genetic factors controlling WH without affecting dormancy (Castonguay et al., 2011; Li et al., 2015). There has been a limited progress in developing non-dormant alfalfa varieties with improved cold and freeze tolerance. Most of the studies have been conducted in Northern latitudes on dormant germplasm or in growth chambers rather than in the field under real winter conditions. Significant differences are known to exist between natural and artificial cold acclimation conditions and therefore plants that are cold acclimated in growth chambers may react differently compared to those acclimated naturally (Dhanaraj et al., 2007). Field grown plants are often exposed to varying light spectrum and intensities compared to the constant conditions in growth chambers. Plants in the field are also frequently exposed to strong winds that influence gene expression and plant structure (Gusta and Wisniewski, 2013). Dhanaraj et al. (2007) documented a large number of genes that were induced in a growth chamber but not under field conditions (Dhanaraj et al., 2007). An understanding of the interconnection between genetic factors and networks that control winter dormancy and WH will provide fundamental knowledge needed for the development of genomic resources that will enable selection of non-dormant alfalfa germplasm that persist well under occasional freezing temperatures. Therefore, dissecting the relationship between alfalfa FD and WH at the genomics level would be valuable to improving alfalfa.

Genetic analysis of FD and WH in alfalfa through QTL mapping requires adequate genome coverage with molecular markers. A large number of SNPs can be obtained cost effectively through next generation sequencing methods like genotyping-by-sequencing (GBS) even in species with no prior genome assemblies. The GBS method developed by Elshire et al. (2011) comprises selective fragmentation of DNA by specific enzymes, ligation of common and barcode adapters, PCR, clean up and sequencing (Elshire et al., 2011). The GBS method has been used successfully in discovering SNP markers in several diploids and autotetraploids crop species such as potato (*Solanum tuberosum* L.), rose (*Rosa hybrida*), and alfalfa (Gar et al., 2011; Li et al., 2014b; Boudhrioua et al., 2017). However, in species with tetrasomic inheritance, only certain biallelic SNPs (simplex, duplex, double simplex) can be mapped. Since most of the mapping software are designed for diploid genomes, mapping autopolyploids is cumbersome. Some new software applications can handle this issue, but they still have limitations. TetraploidMap seems useful in adjusting markers segregating in various ratios (simplex, duplex, double simplex), but it can fit only about 800 markers and works better when each linkage group has <50 markers (Hackett et al., 2007; Li et al., 2014b). Similarly, TetraploidSNPMap can support a higher number of SNPs, but requires SNP dosage data from SNP array (Hackett et al., 2017). However, most of the autotetraploid QTL maps available so far such as potato (da Silva et al., 2017) and alfalfa (Li et al., 2015) maps were constructed using TetraploidMap or TetraploidSNPMap. Mapping autotetraploids with unique kinds of markers using software like JoinMap is also common. Often the pseudo-testcross simplex markers (AB x BB), i.e., markers heterozygous in one parent and not the other, are used to construct autotetraploid genetic maps in software like JoinMap^4^. The pseudo-tetstcross strategy allows the use of several thousand single dose SNPs and is considered a simple method of linkage mapping (Li et al., 2014b). Identifying quantitative trait loci (QTLs) underlying FD and WH will enable understanding the genetic factors controlling these traits and helps in discovering markers associated with each trait. Manipulation of these alleles through MAS will enable the development of non-dormant alfalfa cultivars with improved WH. The objective of this study was to understand the genetic basis of alfalfa FD and WH via genetic linkage analysis and QTL mapping.

## Materials and methods

### Mapping population

An F1 population was developed by crossing a tetraploid dormant (FD = 2) winter-hardy alfalfa cultivar (3010, ♀) with a tetraploid non-dormant (FD = 10) winter susceptible cultivar (CW 1010, ♂). The cross was made in the greenhouse using hand pollination in isolation under 18 hr. light and 6 hr. dark. About 384 F1 seeds were harvested, scarified, inoculated with rhizobium strain N-dure (INTX Microbials LLC, *Sinorhizobium meliloti* and *Rhizobium leguminosarum*), and grown in the greenhouse. In order to confirm the true hybrids, 24 simple sequence repeat (SSR) markers were screened for polymorphism between the parents. These markers were developed from *M*. *truncatula* (Eujayl et al., 2004) and were previously used to genotype tetraploid alfalfa (Li et al., 2011). From the set of 24 SSR markers, three markers with the strongest amplification and highest polymorphic index between the two parents were used to genotype the F1 progeny. Two hundred true F1 hybrids were retained but sufficient numbers of clones for the target locations and replications were obtained only from 184 hybrids. Twenty-four clones per entry were generated through stem cuttings and propagated in the field.

The two parents, 184 F1 progenies, 11 standard check cultivars for FD, and six checks for winter survival^5^ were planted at two locations in Georgia. The first was the J. Phil Campbell Sr. Research and Education Center (JPC) in Watkinsville (33°52′17.8″N 83°27′05.5″W) and the other was the Georgia Mountain Research and Education Center at Blairsville (BVL) (34°50′21.4″N 83°55′20.5″W). The BVL location experiences frequently harsh winters and therefore is considered an ideal location to test alfalfa WH and persistence under cold stress. The average annual precipitation in the BVL location is 55.9 in, the average high temperature in July is 29°C, and the lowest temperature in January is −4°C. At the Watkinsville location, the average annual precipitation is 48 in, the highest temperature in July is 32.2°C while the lowest temperature in January is 0°C. The experimental design at each location was a randomized complete block, with three replications, where four clones from each progeny were planted in a single row plot. Plants were spaced 45 cm within each row, and the rows were spaced 90 cm from each other. Irrigation, fertilization and weed control were applied as necessary.

### Genotyping-by-sequencing (GBS) and marker discovery

Single nucleotide polymorphisms (SNPs) markers were identified using genotyping-by-sequencing of the parents and progeny. DNA of each progeny and parents was extracted using CTAB method with some modifications (Doyle and Doyle, 1987). Alfalfa tissue was collected in 50-ml tubes, freeze dried for 48 h, and grinded using 6–8 zinc-plated copper balls in a Genogrinder (SPEX SamplePrep 2010 Geno/Grinder®) for 6 min at 1,600 rpm. Then, 150 mg of the powder was transferred to 2.0 ml tubes, 900 μl of CTAB buffer was added, vortexed, and the mixture was incubated at 65°C for 1 h. Nine hundred microliter of phenol:chlorofom:isoamyl alcohol (PCI) mix (25:24:1) with pH 5.0 was added to each tube, incubated for 15 min and centrifuged at 12,500 rpm for 15 min and the clear supernatants were pipetted to new 2.0 ml tubes. Equal volume of chloroform:isoamyl alcohol (CIA) mix (24:1) was added to each tube, mixed gently and centrifuged at 12,500 rpm for 15 min. The aqueous upper phase was pipetted into 2.0 ml tube. About 0.6 volumes of chilled isopropanol was added to the tubes and left for 10 min, centrifuged at 13,000 rpm for 20 min. The DNA pellet was washed using 500 μl 70% ethanol, centrifuged at 7,500 rpm for 5 min. The supernatant was discarded, and the DNA pellet was air dried for 1–2 h under the airflow. Then, 100 μl of sterile 10 mM Tris-HCl (pH 8.0) was added and incubated at 4°C for overnight.

The DNA solution was treated with 4 μl (10 mg/ml) of RNase A, followed by 5.0 μl (20 mg/ml) proteinase K, and incubated at 37°C in a water bath for 30 min after each addition. Sterile Millipore grade H_2_O was added (400 μl) and treated with PCI and CIA as described earlier. The supernatant was pipetted into 1.5 ml tubes and 1/10 volume of 3 M Na-acetate pH 5.2 (stored at 4°C) and 2.5 volumes of absolute ethanol was added, left for 10 min and centrifuged for 20 min at 13,000 rpm. The supernatant was discarded and the pellet was washed with 70% ethanol, then air dried. The DNA was dissolved into 50–100 μl of sterile 10 mM Tris-Cl (pH 8.0). High quality DNA was ensured by quantification in Qubit® 3.0 Fluorometer (Thermo Fisher Scientific, Waltham, MA USA) and running DNA samples in 1% agarose gel.

Two GBS libraries were constructed for 184 F1 progenies and the 2 parents. Both libraries were 96-plex including 92 F1 progenies and 2 replications of each parent. The barcode adapters, common adapters, and two PCR primers were ordered from Integrated DNA Technologies (Coralville, IA, USA). The library was constructed using the protocol described in Li et al. (2014b). The DNA samples were digested with methylation sensitive enzyme *ApeKI* and both common as well as barcode adapters were ligated. The step was followed by pooling the libraries (multiplexing) and cleaning up with Qiagen PCR (Qiagen, Germantown, MD) cleanup kit using the protocol provided with the kit. Moreover, the steps were followed by simple PCR using Kapa Library Amplification Readymix (Kapa Biosystems, Wilmington, MA) and two PCR primers. Finally, PCR products were purified using QIAquick PCR purification kit (Qiagen, Germantown, MD). Both libraries were submitted to Georgia Genomics and Bioinformatics Core (GGBC), UGA, for removing short fragments by solid phase reversible immobilization (SPRI), cleanup, and sequencing. Sequencing was performed on an Illumina Next Seq (150 Cycles) 75 PE High Output flow cell with four lanes. The raw sequence data was processed using two pipelines; GBS SNP Calling Reference Optional Pipeline (GBS-SNP-CROP) version 2.0 (Melo et al., 2016) and Tassel 3.0 Universal Network Enabled Analysis Kit (UNEAK) pipelines (Lu et al., 2013) for de novo SNP discovery. These bioinformatics computational steps were performed on in the Unix platform “Zcluster” at the Georgia Advanced Computing Resource Center (GACRC), UGA.

The GBS-SNP-CROP was a useful tool for de novo SNP calling. The raw reads were parsed and trimmed for quality using Trimmomatic software version 0.36 (Bolger and Giorgi, 2014). The trimmed reads were demultiplexed producing high-quality reads for each genotype. The GBS specific mock reference was generated from parsed high-quality reads. The processed reads were mapped to generate standard alignment files using BWA-mem (Santhosh, 1989) and SAMtool version 1.3.1 (Li et al., 2009). Subsequently the SNP master matrix was produced followed by SNP and genotype calling. The raw sequence data was deposited at NCBI SRA website under the accession number SRP150116 and it can be accessed at https://www.ncbi.nlm.nih.gov/sra/SRP150116.

Similarly, UNEAK pipeline was used to process the high quality R1 reads of pair-end data. In UNEAK, the raw R1 reads were filtered, de-multiplexed and trimmed to 64 bp. Similar reads were grouped as a tag, where tags with >10 reads were used for alignment in SNP calling. The parameters used to call and filter the homozygote alleles, heterozygote alleles, minor alleles etc. in UNEAK were as described (Li et al., 2014b). The HapMap output files obtained from UNEAK were processed in Microsoft Excel for separating parental genotypes, removing missing data and testing for segregation ratios using chi-square.

### Linkage map construction

Polymorphic SNPs unique to either 3010 (AB x AA) or CW 1010 (AAx AB) were screened as single dose allele (SDA) markers (Li et al., 2014b). Parental genotypes that were heterozygous (AB) at any replication were considered heterozygous and parental genotype homozygous (AA) in all replications were considered homozygote. Markers that were missing in more than 30% of the progeny were culled. The SDA markers obtained from both pipelines were added and input files were formatted as required by JoinMap 5.0^6^. The SDA segregation ratio (1:1) was confirmed by chi-square tests (*p* > 0.05). The SNPs that were present in more than 30% progenies but have segregation ratios other than 1:1 were considered in segregation distortion (Li et al., 2014b).

Both male and female SDA markers were loaded to JoinMap 5.0 separately. The markers were grouped using minimum independence LOD of 10. The grouped markers were mapped using regression mapping with minimum LOD value of one, maximum recombination frequency of 0.40, and Kosambi mapping function. Linkage maps were generated using Map Chart and the map files were exported. The tags of mapped markers from UNEAK pipeline were separated for each linkage group of both parents. Linkage groups were assigned chromosome numbers using the basic local alignment search tool (BLAST) for querying the consensus tags of SNPs with M. *truncatula* reference genome, *M*. *truncatula* V4.1 genome as described in Li et al. (2014b).

### Phenotypic data analysis and QTL mapping

Alfalfa dormancy data was collected as regrowth height after clipping in the fall and winter. In the fall, canopy height data was taken at 4 weeks after clipping the plants on 21st September according to NAAIC protocol (Teuber et al., 1998). The plant height data at the Watkinsville (JPC) and Blairsville (BVL) locations were taken in subsequent days. The mild winter of 2016/2017 in Georgia allowed taking an early winter and late winter regrowth data. FD was phenotyped in the parents and the pseudo-testcross progeny in fall 2015, fall 2016 and winter (2016/2017). We collected two winter data sets in the season (2016/2017). Because of the mild winter in the Southeastern U.S., it is possible to phenotype seasonal dormancy in field conditions later than northern environments. The regrowth height data was converted to FDR based on the regrowth of 11 standard checks according to NAAIC protocol. FDR of the progeny were assigned based on a regression equation derived from the relationship between standard dormancy ratings of the check cultivars and their regrowth height in each environment. The standard regression lines for each location were established using average dormancy values of three years. The equations were derived for all growing environments and seasons using the Proc Reg procedure in SAS 9.4 (SAS Institute, 2004).

The dormancy phenotypic data consisted of two fall datasets (FD/2015 and FD/2016) for both locations, JPC and BVL, a winter data set collected in the first week of January (referred to as WD/2016 data set, and a second winter data set collected in last week of February (referred to as WD/2017 dataset). WH was evaluated on the F1 population and the two parents on a scale of 0–5 according to NAAIC protocol (McCaslin et al., 2003). Visual scores of winter damage were recorded after each freezing occurrence in winter months. In the case of mild winter, we visually scored plants once a month. Standard checks for winter survival were scored and photographed, and the images were used to guide in the scoring of F1 plants to minimize bias. The visual scores ranged from 1 to 5, where 1 indicates extremely winter-hardy genotypes and 5 indicates non-winter-hardy as described in NAAIC (McCaslin et al., 2003). Phenotypic data for all traits was analyzed using SAS 9.4 (SAS Institute, 2004). The least square (LS) means for all genotypes across environments and within individual environments were estimated for each dataset using PROC GLM (Li et al., 2015). For each trait, a linear additive model was used to perform the analysis of variance (ANOVA) for randomized block design:

$$\begin{array}{lll} \text{Trait value} & = & \text{genotype}+\text{environment}\\ +\text{block }\left(\text{environment}\right)\\ +\text{genotype}*\mathrm{environment}+\mathrm{Error} \end{array}$$

where the trait value refers to the trait phenotypic value estimated by combining the effects of genotype, environment, block, and genotype by environment interaction. The block (environment) was considered random (Haggard et al., 2015). The LS means of all traits for both parents were also obtained within each and across environments. The LS means of the progeny were used as trait value for QTL detection. Pearson correlation coefficients (r) were calculated for both FD and WH trait means within each environment.

QTLs were detected using composite interval mapping (CIM) algorithm on Windows QTL Cartographer version 2.5 (Statistical Genetics, NC State University). The model and parameters used for CIM analysis were as described (Wan et al., 2017). We calculated trait-specific LOD scores using 1000 permutations at genome wide statistical threshold (*P* ≤ 0.05). A QTL was declared significant when the peak LOD value exceeded a conservative LOD threshold of 3.0. In the case of more than one peak, multiple QTLs were declared if LOD values between the peaks falls below 3.0 for more than one contiguous segment for at least one dataset analyzed (Haggard et al., 2015).

The QTL detected in both parents for all traits were classified into two types, stable QTL and potential QTL. The QTL that were detected in more than one season, one environment, or across environments were considered stable QTL. QTL detected either in only one season or one environment were considered as potential QTL. The genomic positions of some major stable QTLs detected for each parental map were indicated on linkage maps using MapChart 2.3 (Voorrips, 2002). The QTL detected for dormancy on the linkage map of the dormant parent, 3010, were given name as “dorm.” Similarly, the QTL detected for non-dormant parent, CW 1010, were named as “ndorm.” The QTL for WH that were detected in the winter-hardy parent 3010 were named as “wh.” The QTL for WH trait detected in the cold susceptible parent (CW 1010) were labeled “ws.” The QTL span was delimited using LOD-1 confidence interval and the QTL were considered identical when the 1-LOD support intervals for QTL overlaps as in previous report (Haggard et al., 2015).

## Results

### GBS and SNPs discovery

A total of 100 Gb raw reads were generated using Illumina NextSeq High Output Flowcell (Illumina, Inc.) amounting to one billion usable paired-reads. Using the GBS-SNP-CROP pipeline for *de novo* SNP calling resulted in 4822 raw SNPs in the pseudo-testcross F1 population. There were 838 single dose allele (SDA) SNPs segregating in the maternal parent 3010 and 794 SNPs segregating in the paternal parent CW 1010. Among these, 423 SDA SNPs from 3010 to 220 SNPs from CW 1010 were filtered as high-quality SDA after chi-square test (α = 0.05) for the segregation ratio of 1:1 (AB: AA). The SNPs obtained from this pipeline were identified with suffix MRG referring “Mock reference genome” followed by SNPs physical position with reference to MRG created using the reads of two parents.

Using the Tassel UNEAK pipeline, 500 million high-quality R1 reads were identified and processed using default parameter settings. A total of 65101 biallelic SNPs were identified. After filtering for missing data (<30%), 34122 (52.4%) SNPs were retained. Additionally, we removed 1625 loci with missing marker information in either parent retaining 32497 SNPs. Therefore, about 50% of the raw SNPs obtained from the UNEAK pipeline were filtered out in the initial screening because of missing data. Among the 32497 SNPs obtained from UNEAK, 4925 SNPs were single dose SNPs for 3010 parent and 2121 SNPs were identified as single dose SNPs for CW 1010 based on chi-square (α = 0.05) test for segregation ratio (1:1).

### Genetic mapping

After merging the GBS SDA-SNP obtained from both Tassel UNEAK and GBS-SNP-CROP pipelines for each parent, we generated a total of 5348 SNPs for the maternal parent 3010 and 2340 SDA for the paternal parent CW 1010 (File S1). Further screening of the SDA loci on JoinMap 5.0^7^ showed that three F1 individuals (ALF107, ALF255 and ALF302) did have several missing loci and were removed from further analysis leaving 181 progenies for mapping. Similarly, 26 loci from 3010 parent to 13 loci of CW 1010 parent were excluded from further analysis because they were identical. Consequently, 5322 SNPs from 3010 to 2327 SNPs from CW 1010 were used in genetic mapping.

**Figure 1:**
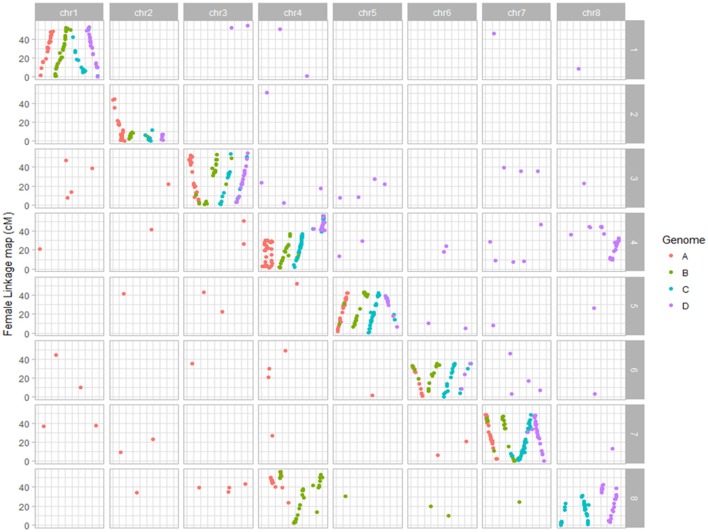
Dot plot displaying the grouping pattern and positions of SNPs on 32 linkage groups of alfalfa 3010 linkage map. Of the 32 groups, each four homologs groups were assigned to a chromosome based on synteny with *Medicago truncatula* genome.

SNPs from both parents were assembled into 32 linkage groups using LOD for independence of 12 or above in the 3010 parent and LOD of 10 or above in CW 1010. Based on the SNP physical positions determined from BLAST analysis, each four linkage groups or haplotypes of each parent were assigned to a corresponding chromosome of *M*. *truncatula* as described in Figure 1 (Li et al., 2014b). Since the majority of the SDA SNPs (92% SDA of 3010 and 90% SDA of CW 1010) were obtained from UNEAK, we only used the SNPs from this pipeline to query the physical locations of markers. The consensus sequence of tag pairs of all mapped SNPs of both parents used to query in BLAST nucleotide.

Thirty-two linkage groups of the maternal parent 3010 consisting of 1837 SDA SNPs were assembled in a linkage map spanning about 2788.4 cM with an average marker density of 1.5 cM/SNP (Table 1, Figure S1). The number of SNPs per linkage group in 3010 parent ranged from 10 to 116. Most of the linkage groups ranged in length from 50 to 100 cM (Table 1, Figure S1). Marker density of the individual LG varied from 0.9 to 5.3 cM/SNP.

**Table 1:** Distribution of SNP markers on 32 linkage groups of each of two alfalfa parents (CW 1010 and 3010).

**Chr^+^**	**Homologs group**	**CW 1010**			**3010**		

		**No. SNPs**	**Length cM**	**MD**^¥^	**No. SNPs**	**Length cM**	**MD**^¥^
1	A	84	56.7	0.7	59	108.5	1.8
1	B	77	73.1	0.9	75	95.1	1.3
1	C	22	71.3	3.2	30	109.6	3.7
1	D	12	40.3	3.4	58	91.6	1.6
2	A	53	114.5	2.2	48	88.9	1.9
2	B	61	78.5	1.3	20	32.7	1.6
2	C	26	76.1	2.9	10	53.3	5.3
2	D	11	73.1	6.6	16	13.6	0.9
3	A	39	70.7	1.8	79	91.9	1.2
3	B	41	50.9	1.2	45	106.7	2.4
3	C	27	62.1	2.3	51	85.9	1.7
3	D	22	26.7	1.2	55	85.7	1.6
4	A	7	48.75	7.0	64	82.1	1.3
4	B	56	121.9	2.2	53	101.2	1.9
4	C	82	72.6	0.9	70	85.5	1.2
4	D	31	71.6	2.3	116	110.4	1.0
5	A	65	88.2	1.4	65	83.1	1.3
5	B	49	84.3	1.7	30	112.3	3.7
5	C	45	47.1	1.0	72	79.2	1.1
5	D	9	51.1	5.7	44	91.2	2.1
6	A	26	55.5	2.1	64	90.3	1.4
6	B	24	42.1	1.8	80	87.9	1.1
6	C	48	91.8	1.9	69	84.9	1.2
6	D	74	91.2	1.2	42	84.2	2.0
7	A	64	83.1	1.3	74	92.3	1.2
7	B	71	46.9	0.7	37	97.3	2.6
7	C	33	82.3	2.5	86	112.1	1.3
7	D	11	42.7	3.9	82	75.9	0.9
8	A	139	28.1	0.2	44	97.9	2.2
8	B	7	46.5	6.6	76	92.1	1.2
8	C	35	66.1	1.9	65	77.8	1.2
8	D	26	71.7	2.8	58	87.2	1.5
Total		1377	2127.55	1.5	1837	2788.4	1.5

*Number of markers, genetic length, and marker density for each homologs group are indicated. The homologs groups (A, B, C, and D) were assigned randomly within each chromosome based on BLAST search result. Chr^+^, Chromosomes; MD^¥^, Marker Density (cM/SNPs)*.

The 32 linkage groups of CW 1010 spanned 2127.5 cM with 1377 mapped markers (Table 1, Figure S2). The average marker density was 1.5 cM/SNP. The number of SNPs mapped on CW 1010 linkage groups varied from 7 to 139 (Table 1, Figure S2). Most of the CW 1010 LG had genetic lengths of 40 to 90 cM. The shortest LG (3D) was 26.7 cM and the longest LG (4B) was 121.9 cM. The individual group marker density in CW 1010 linkage map varied between 0.2 and 6.6 cM/SNP (Table 1).

**Figure 2:**
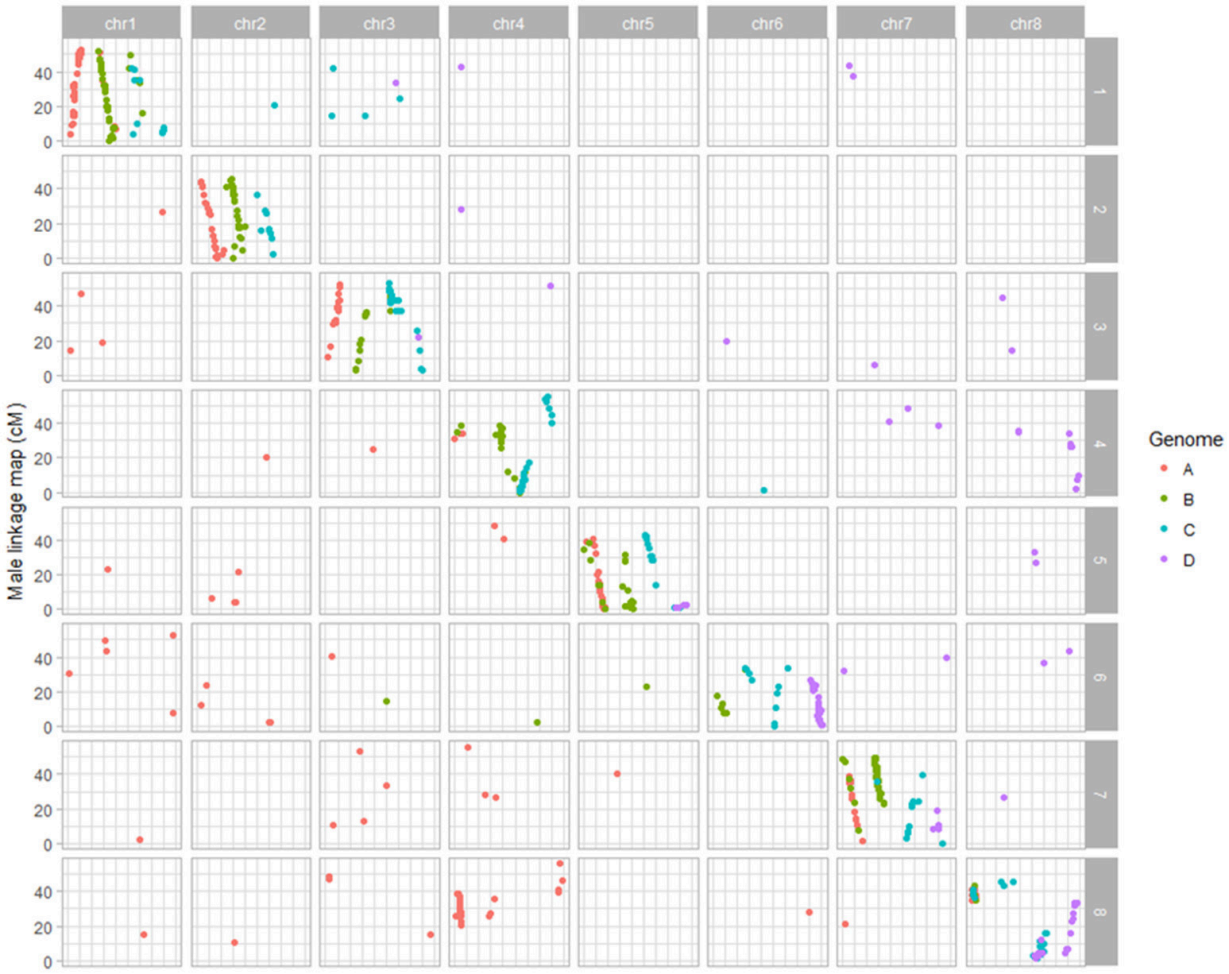
Dot plot displaying the grouping pattern and positions of SNPs on 32 linkage groups of alfalfa CW 1010 linkage map. Of the 32 groups, each four homologs groups were assigned to a chromosome based on synteny with *Medicago truncatula* genome.

BLAST analysis showed that alfalfa genetic loci mapped in this study were syntenic with *M. truncatula* reference genome (Figures 1, 2). From 1837 SNPs mapped in the 3010 parent, 967 (53%) SNPs were aligned to *Medicago* reference genome with 84-100 % identity. On *Medicago* reference genome, 3010 SDA SNPs were aligned within range of 3.3 Kb to 56.4 Mb. The cut-off value used in BLAST analysis ranged from 2.06 E^−06^ to 2E^−26^. Similarly, 741 (53%) SNPs from the parent CW 1010 exhibited similarity with the *M. truncatula* genome with sequence identity of 85 to 100% using the same cut-off value as for 3010 SNPs. CW 1010 SNPs and *M. truncatula* genome similarity were obtained within a range of 0.5 Kb–56.3 MB.

Dot plot maps constructed for each parent using mapped SNPs syntenic to *M. truncatula* clearly displayed the grouping of markers on the 32 LG groups to the corresponding eight *Medicago truncatula* chromosomes (Figures 1, 2; Li et al., 2014b). In the female parent 3010, a translocation of a segment of chromosome four into eight was observed in all four homologs of chromosome eight. Three homologs (4B, 4C, 4D) of chromosome four also possessed segment from chromosome eight, indicating the reciprocal translocation between chromosomes four and eight (Figure 1; Li et al., 2014b). Such translocation was also observed for parent CW 1010, more clearly on haplotypes 4B, 4D, 8A, 8B, and 8D (Figure 2). Several other minor genome rearrangement events such as inversions and other translocations were present in several haplotypes in the maps of both parents. However, this study is focused on marker- trait association rather than structural analysis of the alfalfa genome.

### Phenotypic evaluation and correlation between traits

Eight regression equations were generated to estimate FD of the mapping population at two locations in fall 2015 (FD/2015), fall 2016 (FD/2016), winter 2016 (WD/2016) and winter 2017 (WD/2017). The regression models suggested that the relationship between standard FDR and canopy regrowth height of alfalfa checks was strong and positive. The regression coefficients (*R*^*2*^) ranged from 0.37 for fall 2015 at BVL to 0.73 of winter 2016 dormancy of JPC environment with six out of the eight regression models having coefficient of determinations *R*^*2*^ > 0.50. The regression coefficients of the winter rating were higher than fall ratings at both sites.

There were significant differences between the genotypes (*P* ≤ 0.01) and significant G x E for FD ratings in most dates except for FD/2015 data. Because of the significant G x E, the LS means for each trait were estimated separately for each location (Table 2). The *R*^*2*^ values for each trait, derived from the ANOVA, varied from 0.59 to 0.87 indicating a good fit of the data to the respective linear model for individual tests (Table 2). Dormancy measured in winters (WD/2016 and WD/2017) were highly correlated to each other than the dormancy measured in fall (FD/2015 and FD/2016) in JPC environment (Table 3). The FD trait for winter 2017 rating showed the highest *R*^*2*^ (0.87) and fall 2016 exhibited the least *R*^*2*^ (0.59). The LS means estimated for traits and parents revealed the presence of transgressive segregation on both sides of the parents for both FD and WH traits (Table 2). Some past studies also reported the presence of transgressive segregation in alfalfa pseudo-testcross progeny for such traits (Li et al., 2015).

**Table 2:** Phenotypic means of F1 progeny and parents for FDR and WH scores.

**Trait/Year**	**Location**	**F1 phenotype range (LS means)**	**LS means (3010)**	**LS means (CW 1010)**	**(ANOVA, F1) *R*^*2*^**
FD/2015	JPC	2.3–9.0	6.4	7.5	0.73
FD/2016	JPC	1.9–7.1	4.6	4.7	0.59
WD/2016	JPC	2.0–7.3	2.3	5.7	0.82
WD/2017	JPC	1.2–8.8	2.7	5.3	0.87
FD/2015	BVL	2.2–9.1	5.7	6.6	0.71
FD/2016	BVL	2.89–10.6	4.5	6.5	0.61
WD/2016	BVL	2.5–8.4	4.5	7.9	0.77
WD/2017	BVL	1.6–9.4	4.8	7.5	0.73
FD/2015	JPC & BVL	2.6–8.5	6.1	7.0	0.67
FD/2016	JPC & BVL	3.4–8.0	4.5	5.6	0.62
WD/2016	JPC & BVL	2.3–7.6	3.8	6.8	0.80
WD/2017	JPC & BVL	1.6–8.3	3.7	6.5	0.80
WH/2015	JPC	1–3.2	1	2	0.70
WH/2016	JPC	1.2–5.0	2.5	4.2	0.79
WH/2017	JPC	1–4	1	2.5	0.79
WH/2015	BVL	1–5	2	2.7	0.71
WH/2016	BVL	1–4	1.3	4	0.63
WH/2017	BVL	1–4.9	1.7	4.5	0.70
WH/2015	JPC & BVL	1–3.4	1.5	2.3	0.79
WH/2016	JPC & BVL	1.4–4.1	1.8	4.1	0.78
WH/2017	JPC & BVL	1–4.3	1.4	3.5	0.78

*Coefficient of determination (R^2^) are indicated for each data set. FD, dormancy assessed in fall; WD, dormancy assessed in winter; WH, winter hardiness*.

**Table 3:** Phenotypic correlations (r) among traits based on data collected for Watkinsville (JPC) environment on a pseudo-testcross F1 population (3010 × CW 1010).

	**FD/2015**	**FD/2016**	**WD/2016**	**WD/2017**	**WH/2015**	**WH/2016**	**WH/2017**
FD/2015		0.50^**^	0.62^**^	0.60^**^	0.39^**^	0.52^**^	0.57^**^
FD/2016			0.39^**^	0.43^**^	0.12^NS^	0.31^**^	0.50^**^
WD/2016				0.92^**^	0.22^**^	0.65^**^	0.80^**^
WD/2017					0.23^**^	0.7^**^	0.85^**^
WH/2015						0.16^*^	0.10^NS^
WH/2016							0.68^**^
WH/2017							

*Dormancy was assessed twice in the fall (FD/2015 and FD/2016) and twice in the winter (WD/2016 and WD/2017). The WH data was collected in three consecutive winters (WH/2015, WH/2016 and WH/2017)*.

* *P ≤ 0.05*,

** *P ≤ 0.01, ^NS^non-significant*.

There were significant differences between the genotypes (*P* ≤ 0.01) for WH and significant G x E. Because of the significant G x E, the LS means were estimated for each location in addition to across locations (Table 2). The *R*^*2*^ values for WH, varied from 0.63 to 0.79 indicating a good fit of the data to the respective linear model for individual tests (Table 2). The LS means estimated for F1 progenies and parents revealed the presence of transgressive segregants for WH on both sides of the parents (Table 2).

**Figure 3:**
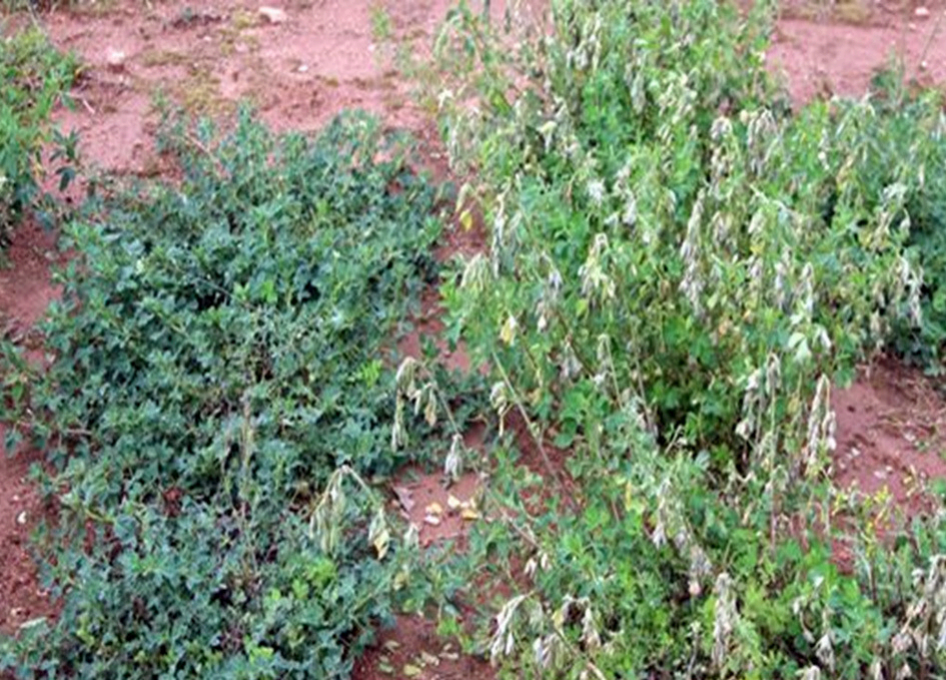
Image showing dormant (left) and non-dormant (right) progeny rows from the pseudo-testcross F1 population (3010 × CW 1010) after frost occurrence in March 2017 at the JPC environment. Frost damage symptoms are clearly visible on the non-dormant progeny.

Pearson's correlation coefficient using trait means showed moderate degrees of correlation between all traits at both locations (Tables 3, 4). Overall, there were stronger positive correlations between dormancy and WH when dormancy was assessed in winter compared to fall assessment (Figure 3, Tables 3, 4). In the JPC environment, the coefficient of correlations between dormancy rating and WH ranged from 0.12 to 0.57 when dormancy was assessed in the fall, while it ranged from 0.22 to 0.85 when dormancy was assessed in winter (Table 3). The same trend was observed in the BVL location. The coefficient of correlations between dormancy rating and WH ranged from 0.16 to 0.50 when dormancy was assessed in the fall, while it ranged from 0.22 to 0.57 when dormancy was assessed in winter (Table 4).

**Table 4:** Phenotypic correlations among traits based on data collected at the BVL location on a pseudo-testcross F1 population (3010 × CW 1010).

	**FD/2015**	**FD/2016**	**WD/2016**	**WD/2017**	**WH/2015**	**WH/2016**	**WH/2017**
FD/2015		0.42^**^	0.6^**^	0.58^**^	0.16^*^	0.16^*^	0.33^**^
FD/2016			0.6^**^	0.64^**^	0.25^**^	0.43^**^	0.50^**^
WD/2016				0.92^**^	0.24^**^	0.27^**^	0.57^**^
WD/2017					0.22^**^	0.25^**^	0.51^**^
WH/2015						0.34^**^	0.46^**^
WH/2016							0.54^**^
WH/2017							

*Dormancy was assessed twice in the fall (FD/2015 and FD/2016) and twice in the winter (WD/2016 and WD/2017). Winter hardiness (WH) data were collected in three consecutive winters (WH/2015, WH/2016 and WH/2017)*.

* *P ≤ 0.05*,

** *P ≤ 0.01, ^NS^non-significant*.

### QTL mapping of FD and WH

Within the 32 homologs of the 8 alfalfa chromosomes, we detected 45 significant (*P* ≤ 0.05) QTLs for FD and 35 QTLs for WH on both male and female linkage maps (Tables 5–8). Most of the QTLs detected using phenotypic data across environments matched QTLs detected for individual environments with slight variation in their LOD magnitude and interval. Seven QTLs for dormancy and three QTLs for WH detected across environments were exclusively different from QTLs detected for individual environments indicating a potential effect of G x E on trait values (Table 8).

**Table 5:** Stable QTLs for alfalfa FD detected in an F1 (3010 × CW 1010) pseudo-testcross population based on phenotypic data assessed in fall and winter at two locations.

**Parent**	**QTL code**	**Chr**	**Year/Location**	**Peak markers**	**Peak LOD**	**R^2^**	**Allele dir**.	**LSI (cM)**	**Flanking markers**
3010	dorm1	1A	(π1), π4, β2, $1, $2	TP995	10.9	0.16	(−)	90.6–92.9	TP995–TP78651
3010	dorm2	1A	π1, π3, (π4), β3, β4, $1, $4	TP86274	6.2	0.11	(−)	98.2–104	TP65855–TP86274
3010	dorm3	7A	π1, (β3), β4, $1	TP24733	6.2	0.11	(−)	36.9–38.8	TP55743–TP34483
3010	dorm4	4C	(π1), β1, $1	TP56893	7.5	0.11	(−)	58.6–61.0	TP55689–TP56893
3010	dorm5	7B	(π4), β3	TP31689	4.1	0.08	(−)	34.6–48.7	TP31689–TP33803
3010	dorm6	7A	(π1) π3, π4, β3, β4, $1, $3	TP69889	5.5	0.07	(−)	47.3–52	TP59349–TP71458
3010	dorm7	3A	β1, β2, (β3)	TP32327	3.8	0.06	(−)	25.8–26.3	TP3895–TP54529
CW 1010	ndorm1	8D	(π1) π3, π4, β1, $1	TP2543	9.9	0.13	(+)	44.6–46.3	TP2543–TP88682
CW 1010	ndorm2	7C	π3, (π4), β3	TP38417	9.0	0.12	(+)	46.9–51.1	TP38417–TP54614
CW 1010	ndorm3	5B	(β1), $1	MRG_32692305	4.4	0.08	(+)	15.2–17.9	TP79886–MRG_32692305
CW 1010	ndorm4	8D	π3, (π4), β1, $3	TP24024	6.1	0.07	(+)	53.7–54.8	TP24024–TP25406
CW 1010	ndorm5	1B	π1, (β4), $4	TP57411	5.1	0.07	(+)	14.2–15.3	TP63551–TP32288
CW 1010	ndorm6	5B	(π1), β2	TP26770	5.5	0.06	(+)	48–49.5	MRG_37364973–TP26770
CW 1010	ndorm7	8D	π3, (π4)	TP67491	3.9	0.06	(+)	63.9–65.4	TP67491–TP71707
CW 1010	ndorm8	1B	π1, π3, β1, (β4), $4	TP41786	3.2	0.05	(+)	21.1–23.7	TP83000–TP7086
CW1011	ndorm9	1B	(π3), π4, β3	TP52371	4.2	0.05	(+)	31.9–33.8	TP35547–TP23336
CW 1010	ndorm10	7B	π3, (π4)	TP14107	3.8	0.04	(+)	23.3–24.2	TP14107–TP9019
CW 1010	ndorm11	7B	π3, (π4)	TP7325	3.1	0.03	(+)	11.7–12.7	TP32866–TP42483

*Seven QTLs for 3010 and eleven QTLs for CW 1010 were mapped on respective genetic linkage maps for phenotypic datasets of more than one environment and/or year*.

*The symbol with bracket in the column “year/location” indicates the source dataset from which the other parameters in the same row were pulled*.

*JPC environment dormancy data for the period: π1 = 2015, fall; π2 = 2016, fall; π3 = 2016, winter; π4 = 2017, winter*.

*BVL environment dormancy data for the period: β1 = 2015, fall; β2 = 2016, fall; β3 = 2016, winter; β4 = 2017, winter*.

*Across environment dormancy for the period: $1 = 2015, fall; $2 = 2016, fall; $3 = 2016, winter; $4 = 2017, winter*.

*Chr., Chromosome; Dir., Direction; LSI, 1-LOD support interval in cM unit*.

**Table 6:** Stable QTLs for alfalfa WH identified in an F1 (3010 × CW 1010) pseudo-testcross population based on phenotypic data assessed in three consecutive winters at two locations.

**Parent**	**QTL code**	**Chr**.	**Year/Location**	**Peak marker**	**Peak LOD**	**R^2^**	**Allele dir**.	**LSI (cM)**	**Flanking markers**
3010	wh1	1A	(λ2), λ4, ϕ3	TP995	7.1	0.13	(+)	90.8–93.2	TP995–TP6492
3010	wh2	7A	(λ3), ψ1, ϕ1	TP24733	7.1	0.12	(+)	37.5–39.0	TP55743–TP34483
3010	wh3	1A	(λ2), ϕ3	TP65855	5.4	0.11	(+)	98.2–104.5	TP65855–TP86274
3010	wh4	8A	(ψ2), ϕ2	TP10810	5.1	0.09	(−)	74.1–74.8	TP58070–TP10810
3010	wh5	3A	(ψ3), ϕ3	TP71671	5.1	0.09	(+)	47.6–50.1	TP52425–TP67563
3010	wh6	1C	(λ1), ψ1, ϕ1	TP37162	4.1	0.07	(+)	96.3–99	TP37162–TP57104
3010	wh7	7A	(λ1), λ3, ϕ1	TP58371	3.2	0.07	(+)	26.4–29.3	TP58371–TP34795
3010	wh8	4C	(λ2), ψ1, ϕ1	TP2323	3.5	0.06	(+)	27.1–31.1	TP6532–TP4218
CW 1010	ws1	7C	λ2, (λ3), ψ1	TP54614	9.8	0.14	(−)	48.4–51.4	TP38417–TP54614
CW 1010	ws2	8D	(λ3), ψ3, ϕ3	TP52817	7.5	0.10	(−)	40.8–43.6	TP52817–TP46951
CW 1010	ws3	7A	(ψ3), ϕ2	TP71946	5.7	0.10	(+)	8.6–17.6	TP78230–TP71946
CW 1010	ws4	7A	ψ2, ϕ2	TP81779	5.3	0.10	(+)	31–32.1	TP16325–TP70376
CW 1010	ws5	8D	(λ2), ϕ3	TP2543	5.2	0.08	(−)	44.6–45.8	TP2543–TP6748
CW 1010	ws6	7B	(λ3), ψ1, ϕ1	TP87913	5.6	0.07	(−)	35.9–37	TP87913–TP85708
CW 1010	ws7	7B	(λ3), ψ1, ϕ3	MRG_41805356	4.9	0.07	(−)	24.5–25.3	TP49165–TP74211
CW 1010	ws8	8D	(λ1), λ2	TP8426	3.3	0.06	(−)	58.6–63.1	TP69982–MRG_7512818
CW 1010	ws9	1A	(λ3), ϕ3	TP40020	3.4	0.04	(+)	47.2–48	TP60690–TP40020

*Eight QTLs from 3010 to 9 QTLs from CW 1010 were mapped on respective genetic linkage maps using phenotypic datasets of more than one environment and/or year. The symbol with bracket in the column “year/location” indicates the source dataset from which the other parameters in the same row were generated*.

*JPC environment winter hardiness data for the period: λ1 = 2015; λ2 = 2016; λ3 = 2017*.

*BVL environment winter hardiness data for the period: ψ1 = 2015; ψ2 = 2016; ψ3 = 2017*.

*Across environment winter hardiness data for the period: ϕ1 = 2015; ϕ2 = 2016; ϕ3 = 2017*.

**Table 7:** Potential QTLs for dormancy and WH identified in an F1 pseudo-testcross (3010 × CW 1010) population.

**Trait**	**Parent**	**QTL code**	**Chr**.	**Year/Location**	**Peak marker**	**Peak LOD**	**R^2^**	**Allele dir**.	**LSI (cM)**	**Flanking markers**
FD	3010	dorm8	1A	β1	TP73186	7.7	0.12	(−)	69.1–71.3	TP73186–TP70400
FD	3010	dorm9	5A	π1	MRG_28485316	5.3	0.08	(−)	36.1–45.8	MRG_28485316–TP63204
FD	3010	dorm10	6D	π3	MRG_2402742	4.7	0.08	(−)	16.7–24.0	MRG_2402742–TP18699
FD	3010	dorm11	4D	π2	TP64707	4.5	0.08	(−)	101.3–101.9	TP64707–TP61536
FD	3010	dorm12	1C	π2	TP78612	3.4	0.08	(−)	62–70.3	TP78612–TP68882
FD	3010	dorm13	7B	β4	TP43449	4.2	0.07	(−)	15.4–18.5	TP14416–TP25746
FD	3010	dorm14	2C	π2	TP29084	3.8	0.07	(−)	32.7–39.5	TP29084–TP82709
FD	3010	dorm15	4C	π4	TP64526	4.2	0.06	(−)	16.3–18.8	MRG_18042076–TP64526
FD	3010	dorm16	2B	π3	TP69826	4.0	0.06	(−)	25.3–28.7	TP78664–TP59834
FD	3010	dorm17	5A	β1	TP63107	3.8	0.06	(−)	24.1–27.6	TP89078–TP46688
FD	3010	dorm18	3A	β1	TP32175	3.3	0.05	(−)	9–15	TP32175–TP44970
FD	3010	dorm19	3D	π3	TP67190	3.2	0.05	(−)	5.6–13.3	TP67190–TP58690
FD	3010	dorm20	3A	β2	TP32136	3.1	0.05	(−)	18.6–22	TP48316–MRG_4754683
FD	3010	dorm21	7D	β4	TP32437	3.1	0.05	(−)	5.2–7.7	TP79530–TP53493
FD	3010	dorm22	7C	π2	MRG_30285700	3.1	0.05	(−)	19.9–22.4	MRG_30285700–TP49176
FD	3010	dorm23	5D	π1	TP31552	3.2	0.04	(−)	36.5–41.6	TP31552–TP28126
FD	CW 1010	ndorm12	4D	β4	TP32802	6.7	0.12	(+)	64.5–66.1	TP32802–TP5506
FD	CW 1010	ndorm13	7A	β2	TP63954	5.9	0.10	(−)	28.1–29.4	TP63954–TP87998
FD	CW 1010	ndorm14	4B	β4	TP11836	3.8	0.05	(+)	71.2–72.4	TP11836–TP10328
FD	CW 1010	ndorm15	7A	β1	MRG_31595966	3.2	0.04	(+)	60.1–61.4	MRG_7180813–TP51152
WH	3010	wh9	7C	ψ2	TP84244	4.9	0.09	(−)	72.7–73.9	TP84244–TP44147
WH	3010	wh10	7C	λ1	TP74326	4.5	0.09	(+)	106.5–110.6	TP74326–TP30485
WH	3010	wh11	8B	ψ3	TP34659	3.9	0.08	(+)	43.3–47	TP24160–TP34659
WH	3010	wh12	8D	ψ3	TP15842	3.7	0.07	(+)	77–80.1	TP33611–TP15842
WH	3010	wh13	3B	λ1	TP63723	3.2	0.06	(+)	44.2–50.3	TP63723–TP46610
WH	3010	wh14	3D	ψ2	TP26775	3.3	0.06	(+)	28.5–33.6	TP88373–TP16429
WH	3010	wh15	2B	λ1	TP6025	3.1	0.05	(+)	8.7-11.1	TP19047–TP6025
WH	3010	wh16	2C	λ3	TP29084	3.2	0.05	(+)	33.5–40	TP29084–TP82709
WH	CW 1010	ws10	4D	λ1	TP88199	10.1	0.18	(−)	33.9–37.8	TP54779–TP88199
WH	CW 1010	ws11	1B	ψ2	TP66690	3.8	0.076744	(+)	41.8–43.1	TP64641–TP66690
WH	CW 1010	ws12	5A	ψ3	TP33164	3.9	0.066623	(−)	62.7–63.6	TP33164–TP30048
WH	CW 1010	ws13	1B	ψ2	TP7086	3.1	0.068061	(−)	22.9–24.1	TP7086–TP65701
WH	CW 1010	ws14	8A	ψ3	MRG_12811807	3.3	0.055791	(−)	20.1–20.3	MRG_12811807–TP29734
WH	CW 1010	ws15	1A	λ3	TP81842	3.7	0.047139	(+)	54.6–55.8	TP6332–TP81842
WH	CW 1010	ws16	6A	λ3	TP60069	3.4	0.044139	(−)	52.5–54.7	TP60069–TP5275

*These QTLs were detected only for a single location and a single year. The symbols used in this table have exactly same abbreviations as given for Tables 5, 6*.

**Table 8:** Potential QTLs for dormancy and WH identified in a pseudo-testcross F1 population (3010 × CW 1010) using phenotypic data generated across two environments (JPC and BVL).

**Trait**	**Parent**	**QTL code**	**Chr**.	**Year**	**Peak marker**	**Peak LOD**	**R^2^**	**Allele dir**.	**LSI (cM)**	**Flanking markers**
FD	3010	dorm24	3A	$4	TP67563	5.4	0.09	(−)	49.4–51.8	TP71671–TP76041
FD	3010	dorm25	3D	$4	MRG_38650252	3.6	0.09	(−)	70.1–74.4	MRG_31477229–TP57603
FD	3010	dorm26	3D	$2	TP16817	4.3	0.09	(−)	76.3–79.5	TP16817–TP5092
FD	3010	dorm27	1C	$1	TP32721	4.8	0.07	(−)	81.8–85.1	TP32721–TP40620
FD	3010	dorm28	1A	$3	TP46942	3.6	0.06	(−)	72–73	TP72089–TP73780
FD	CW 1010	ndorm16	4D	$3	TP69818	3.1	0.09	(+)	8.3–10.7	TP69818–TP82286
FD	CW 1010	ndorm17	4D	$2	TP80681	3.9	0.08	(+)	19.7–23.5	TP80681–TP83595
WH	3010	wh17	4C	ϕ1	TP81375	4.6	0.10	(+)	37.6–39.5	TP80478–TP81375
WH	3010	wh18	3D	ϕ3	TP43991	5.1	0.09	(+)	58.9-63.2	TP50808–TP87713
WH	3010	wh19	7A	ϕ3	TP71458	3.7	0.06	(+)	50.5–52	TP15177–TP71458

Across environment dormancy:

*$1 = 2015, fall; $2 = 2016, fall; $3 = 2016, winter; $4 = 2017, winter*.

Across environment winter hardiness:

*ϕ1 = 2015; ϕ2 = 2016; ϕ3 = 2017*.

*Chr., Chromosome; Dir., Direction; LSI, 1-LOD support interval in cM unit*.

### Fall dormancy (FD)

**Figure 4:**
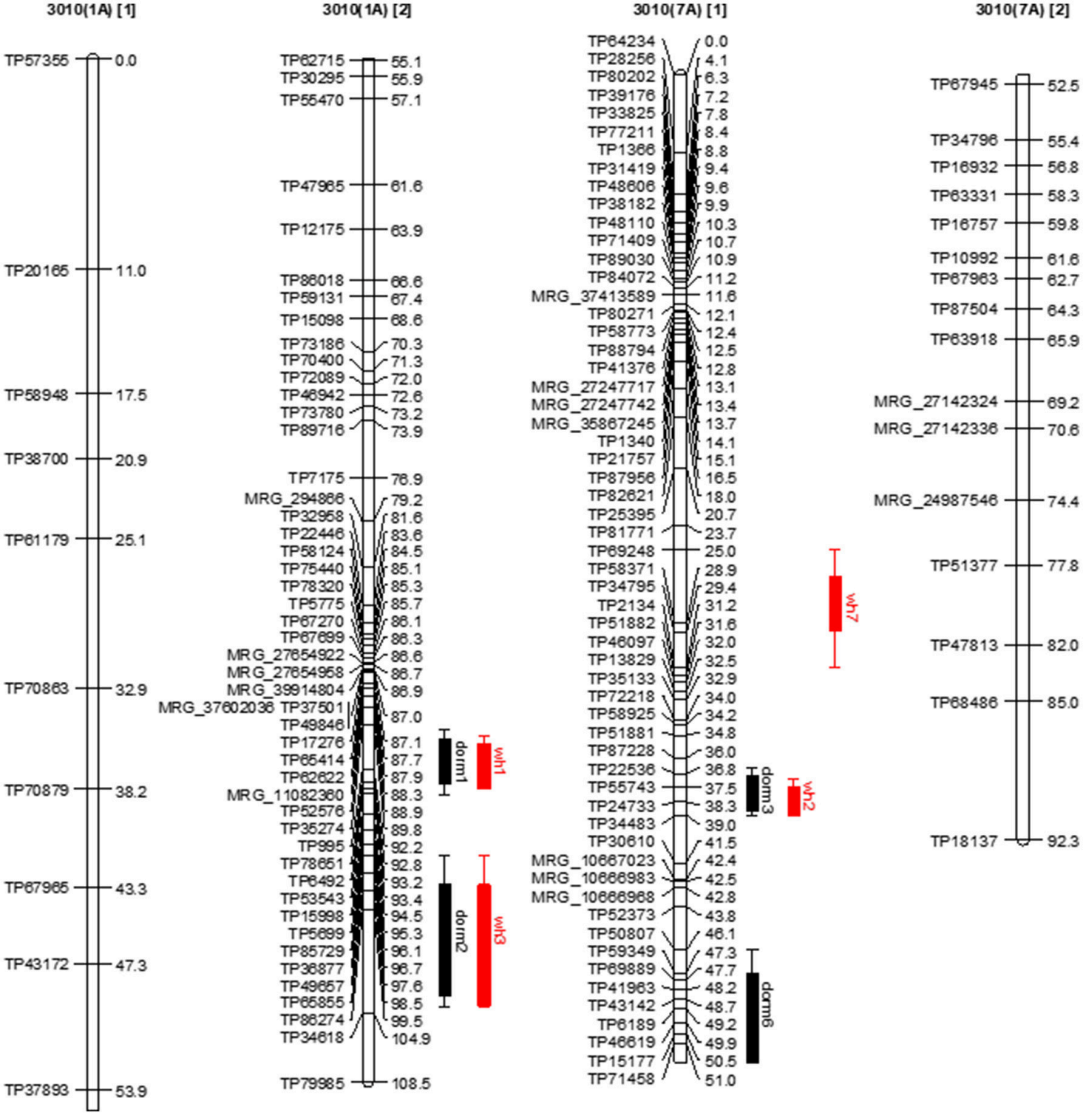
Dormancy (black bar) and WH (red bar) stable QTLs mapped on linkage maps of homolog 1A **(left)** and 7A **(right)** for 3010 parent. The QTL bars have two intervals, an inner (1-LOD support) interval and an outer (2-LOD support) interval, where the rectangle represents inner interval and the line represents the outer. Some stable QTLs for dormancy were co-localized with WH in the same genomic regions.

Seven stable QTL for FD were identified in the dormant parent 3010. These QTLs were consistently and repeatedly detected across data sets within overlapping 1-LOD support intervals (Table 5, Figure 4). The seven dormancy QTL for 3010 (dorm1, dorm2, ……, dorm7) were detected on homologs 1A, 3A, 4C, 7A and 7B. Another 21 potential QTLs (dorm8, dorm9,….…, dorm28) were detected in various homologs of 3010 chromosomes: 1, 2, 3, 4, 5, 6, and 7 (Tables 5, 7, 8). Five of the seven stable QTLs were detected also across environments. The most important dormancy QTL (dorm1) for 3010 parent (*R*^*2*^ = 0.16) was detected on homolog 1A and was located at 90.6–92.9 cM. The same homolog harbors another QTL (dorm2) with a LOD value of 6.2 and a peak at the interval 98.2–104 cM (Table 5, Figure 4). Besides these two stable QTLs for 3010 parent, other potential QTLs (dorm 8, dorm 12, dorm 27 and dorm 28) were detected on homologs of 3010 chromosome 1, suggesting that this chromosome is important for the dormancy trait.

Further, dorm1 and dorm2 QTLs of 3010 parent mapped on homolog 1A were located within similar genomic location of alfalfa fall height QTLs (92a and 104a) in the WISFAL-6 cultivar reported previously (Li et al., 2015). (Li et al., 2015) mapped fall height on eight alfalfa linkage groups assigned using eight *M*. *truncatula* chromosomes. Unlike 3010 QTLs dorm1 and dorm2 detected in our study, WISFAl-6 dormancy QTL of LG1 had positive effect on trait value because the source parent WISFAL-6 had higher FD levels (Li et al., 2015).

Another two stable dormancy QTLs from 3010 were detected on chromosome 7A (*R*^*2*^ = 0.07–0.11). Homologs of this chromosome also harbor QTL dorm3, dorm5, dorm6, and dorm13 (Tables 5, 7). The QTL dorm6 also falls within similar genomic regions of a fall height QTL in LG 7 as reported previously (Li et al., 2015). Two other potential QTL on homologs of this chromosome at LOD = 3.1 include dorm21 and dorm22 that were located on two homologs (7D and 7C) of 3010 chromosome7. Further, the homologs of 3010 chromosomes: 3, 4 5, 6, and 2 also harbored significant (*P* ≤ 0.05) QTLs for dormancy (Tables 5, 7). All dormancy QTLs detected on 3010 parent had negative effects on trait value since the parent was a dormant type.

In the CW 1010 parent, 11 stable QTLs and six potential QTLs for FD were detected. All the stable QTLs for CW 1010 were detected on homologs of the chromosomes 1, 7, 5, and 8 (Table 5). The CW 1010 chromosome 8 exhibited a broader QTL peak extending from ~44 to ~ 66 cM. However, there is the possibility of presence of more than one QTL in the region because of decreasing LOD value between multiple QTL peaks. Therefore, we reported three different stable QTLs for this region to ensure the accuracy of QTL and corresponding phenotypic values of markers in the region. A past study (Li et al., 2015) also reported a QTL (46a) positively affecting fall plant height in the same genomic region (40–56 cM) of LG 8 of the alfalfa cultivar ABI408 providing more supportive evidence for this QTL. The QTL (ndorm1) from CW 1010 with positive effect on the trait value (*R*^*2*^ = 0.13) was detected on homolog 8D at the interval 44.6–46.3 cM (Table 5). Other stable QTLs for dormancy detected from CW 1010 parent including ndorm2, ndorm3, ndorm4 and ndorm6, shared common genomic regions with QTLs for fall height reported previously (Li et al., 2015). However, two CW 1010 dormancy QTLs, ndorm3 and nodorm6, had contrasting effects in trait value than previously reported QTLs of the corresponding linkage groups. Of 17 total CW 1010 dormancy QTLs, 16 QTLs had additive effect in favor of trait value and one potential QTL (ndorm13) had negative effect on trait value (Table 7).

Although we detected dormancy QTLs for both 3010 and CW 1010 parents in most of the datasets, a higher number of stable QTLs were repeatedly detected using winter dormancy data compared to fall data of all environments (Tables 4, 5, 7). For the 3010 parent, only two stable QTLs were observed for each 2015 and 2016 fall datasets of BVL location, while two and four stable QTLs were detected for JPC winter datasets WD/2016 and WD/2017, respectively (Table 5). Only two stable dormancy QTLs for 3010 parent (dorm1 and dorm4) were repeatedly detected in fall. However, four stable dormancy QTLs (dorm2, dorm3, dorm5 and dorm6) were repeatedly detected in more than one winter data (Table 5). For CW 1010 parent, out of 11 stable dormancy QTLs, only six stable QTLs were identified for all FD data of both locations, and nine stable QTLs were detected for winter datasets of both locations (Table 5). Five and three potential QTLs were detected only in cross environments analysis for both parents 3010 and CW 1010 indicating the presence of G x E (Table 5). There was also slight shift of QTL peaks identified in across environments compare to those QTL identified in individual environment data sets.

### Winter hardiness (WH)

Eight stable and 11 potential QTLs were identified from the 3010 parent for WH trait (Tables 6–8). The stable QTLs (wh1, wh2, ……, wh8) were detected on homologs of chromosomes: 1, 3, 4, 7, and 8, and 11 potential QTLs (wh9, wh10, ……, wh20) were detected on homologs of chromosomes: 2, 3, 4, 7 and 8. The QTL wh1 on homolog 1A (position 90–93.2 cM) has the largest *R*^*2*^ (0.13) followed by QTL wh2 on homolog 7A (0.12) and wh3 on homolog 1A (0.11) (Table 6). The wh1 QTL was located in the same genomic region of previously identified QTL (100a) in WISFAL-6 alfalfa, but with opposite effect (Li et al., 2015). Similarly, other two potential QTLs, wh10 on homolog 7C and wh16 of homolog 2C were also detected within similar genomic locations of previously identified QTLs for winter injury for ABI408 (LG7, 109a) and WISFAL-6 cultivar (LG2, 36b), respectively (Li et al., 2015). With the exceptions of a stable QTL (wh4) on homolog 8A and one potential QTL (wh9) on homolog 7C, which had negative effects (−) on WH, all other WH QTLs detected on 3010 possessed positive effects (+) on WH (Tables 6–8).

Sixteen (nine stable and seven potential) QTLs for WH were detected for the winter susceptible parent CW 1010 and were coded as (ws1, ws2, …, ws16) (Tables 6, 7). Major stable QTLs for WH in CW 1010 were detected on homologs of chromosomes 1, 7 and 8. The QTL ws1 had the highest *R*^*2*^ (0.14) followed by ws2, ws3, and ws4 (*R*^*2*^ = 0.10). However, contrary to ws1 and ws2, the ws3 and ws4 QTLs had positive effects (+) on WH (Table 6). The three stable QTLs, detected for WH trait on homolog 8D, appeared in a single span for JPC data. However, for BVL and across environments, the QTL on 8D was separated into three different QTLs and were reported as such. Of the total 16 WH QTLs in CW 1010, ws12 on homolog 5A and ws16 on homolog 6A were detected within similar genomic regions reported previously for winter injury in the cultivars WISFAL-6 and ABI408 (Li et al., 2015). Most of the WH QTLs for CW 1010 have negative effects (−) on the trait except QTL ws3, ws4, ws9, ws11 and ws15 (Tables 6, 7).

### Association between dormancy and winter hardiness

Among the seven stable dormancy QTLs detected in the 3010 the dorm1 and dorm2 on homolog 1A shared the same genomic location as WH stable QTLs wh1 and wh3, respectively (Figure 4). Similarly, the dormancy QTL dorm3 overlapped with WH QTL wh2 on 7A with < 1 cM shift (Figure 4). Another stable QTL dorm6 also shared the same genomic location with a potential winter hardiness QTL wh20 on 7A. Three stable QTLs (dorm4, dorm5, and dorm7) in the 3010 parent were unique and located on different chromosomes than winter hardiness. Of the 21 potential dormancy QTLs in the 3010 parent, except dorm14 and dorm24, other 19 were also located in different genomic regions from the QTLs of WH (Tables 5–8). Therefore, of the 28 dormancy QTLs detected in 3010 parent, 22 QTLs were located in separate genomic positions than the QTLs of WH indicating differences of two traits at the genomic level.

**Figure 5:**
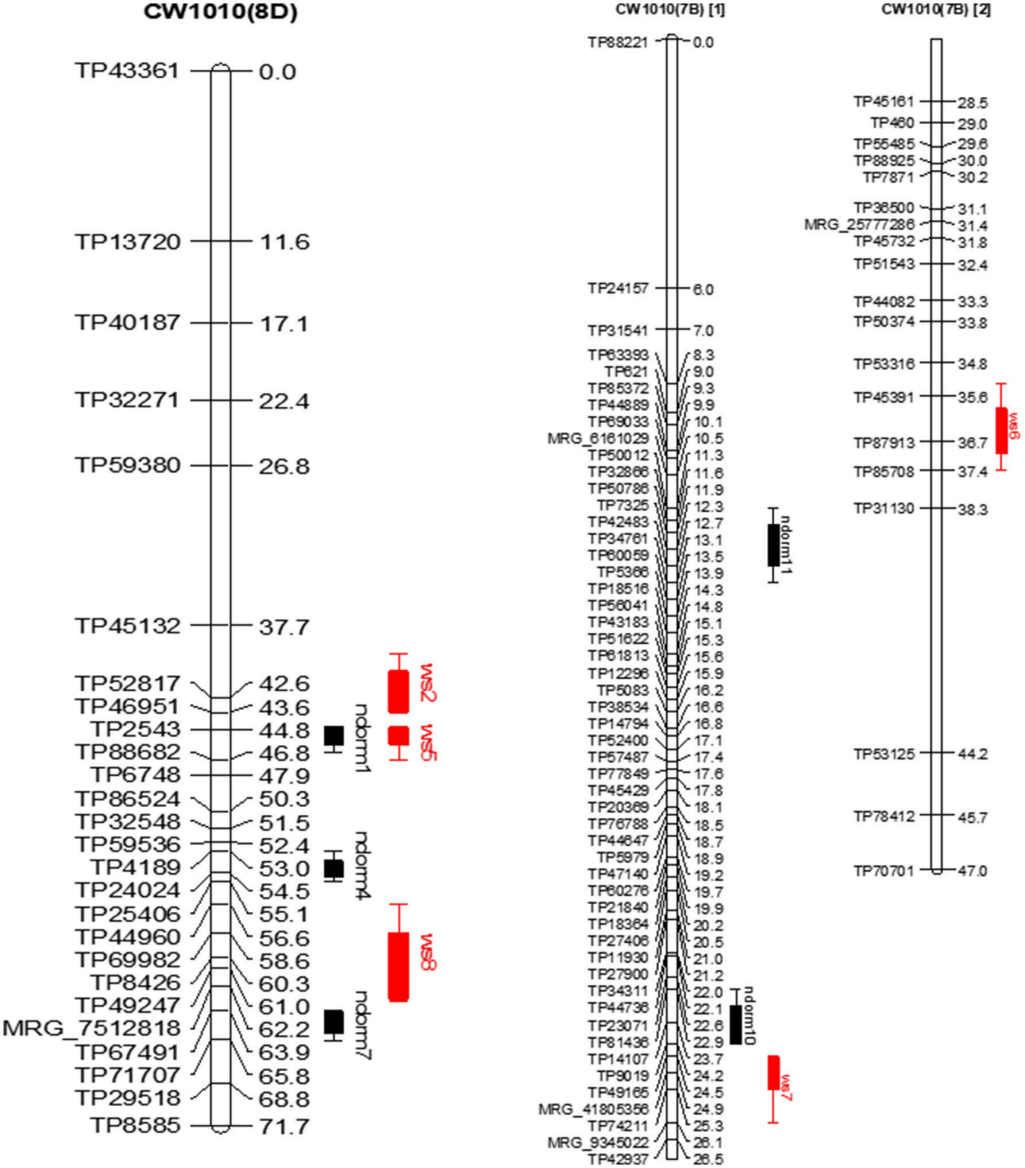
Dormancy (black bar) and WH (red bar) stable QTLs mapped on linkage maps of homolog 8D **(left)** and 7B **(right)** for CW 1010 parent. The QTL bars have two intervals, an inner (1-LOD support) interval and an outer (2-LOD support) interval, where the rectangle represents inner interval and the line represents the outer. Some stable QTLs for dormancy were co-localized with WH in the same genomic regions.

In the CW 1010 parent, the stable QTLs ndorm1 and winter hardiness QTL ws5 shared the same genomic location on homolog 8D (Figure 5). Likewise, the QTL ndorm2 and ws1 also reside on same position on homolog 7C. Another stable dormancy QTL of CW 1010 ndorm8 shares genomic location with a potential QTL for winter hardiness ws13 on homolog 1B. Moreover, a stable dormancy QTL ndorm7 and a winter hardiness QTL ws8 were located at nearby positions on the homolog 8D (Figure 5). Similarly, ndorm10 and ws7 also shared partial genomic position on homolog 7B of CW 1010 (Figure 5). Other stable dormancy QTLs from the parent CW 1010 such as ndorm3, ndorm4, ndorm5, ndorm6, ndorm9, and ndorm11 were located in separate genomic positions than those QTLs for WH. All potential QTL detected for CW 1010 for dormancy as well as for WH were also located in distinct genomic regions (Tables 5–8). Therefore, of the 17 dormancy QTLs detected in CW 1010 parent, 12 QTLs were located in separate genomics regions than the QTLs for WH (Tables 5–8).

## Discussion

### Segregation and phenotypic relationship between traits

The regrowth height and dormancy ratings of the NAAIC standard checks used in this study showed a better fit to the regression model in winter height data with *R*^*2*^ up to 0.73 compared to the height data taken in fall (*R*^*2*^ ~ 0.50) (Tables 3, 4). The fall data is collected around the third week of October according to NAAIC protocol. In southern environments, temperatures at this time of the year are still very favorable for active growth of alfalfa. Fall 2016 was a very unusual season in Georgia because of the historical drought in the region (Adhikari et al., 2018), which led to a very limited growth and erratic regrowth after clipping in both experimental sites. This could be the main reason that the two parents did not exhibit differences in their heights for this season (Table 2) and a few QTLs were detected based on the 2016 fall data. The positive *R*^*2*^ for the regression of standard checks regrowth height data on their FDR for all seasons, suggests that that determining dormancy of alfalfa genotypes using regrowth height after clipping is a reliable approach.

The non-dormant parent CW 1010 exhibited slightly lower dormancy level (4.7–7.9), than it supposed to be in most of the years. The 3010 parent mostly exhibited the expected dormancy level between 2.3and 6.4 (Table 2). Such fluctuations of estimated dormancy level from their standard dormancy are primarily due to environmental and seasonal variations. The largest deviations from the standard dormancy ratings of the parents were observed in the data collected in fall season, suggesting that rating alfalfa dormancy in the Southeastern U.S.A based on regrowth height after clipping in third week of September is not reliable. Previous reports also suggested that FD assignment should be done in sites where the cultivars are broadly adapted (Teuber et al., 1998). Dormancy assessment in winter months showed a better approximation of the expected values with less variation compared to the fall assessment. Winter assessed dormancy also showed a better repeatability in both locations as suggested by the high correlation between WD/2016 and WD/2017 (*r* = 0.92, *p* < 0.01) (Tables 3, 4). The LS means of dormancy ratings of the F1 progeny varied from 1.2 to 10.6 and showed transgressive segregation around the parental values (Table 2).

WH rating scores for 3010 parent varied from 1.4 to 1.5 across locations, which is within the range of the known score 2 for this cultivar. For the non-winter-hardy parent CW 1010, the scores varied between 2.3 and 4.1 across locations (Table 2). The F1 progeny also varied in their WH level with the largest differences observed in 2017 (WH/2017) at BVL location. Transgressive segregants were observed for both dormancy and WH similar to previous alfalfa reports (Li et al., 2015). This suggests the presence of complementary alleles for both traits in each of the parents (Li et al., 2015). The moderate positive correlations (*r*) observed between dormancy and WH irrespective of the time of assessment is a clear indication of the weak association between the two traits (Tables 3, 4).

### Genetic linkage map

Genetic maps are important tools for genetic analysis of quantitative traits through QTL and comparative mapping. They are also useful for genome assembly and marker development for MAS (Moriguchi et al., 2012; Li et al., 2014b). In alfalfa, genomic resources are very scarce and even though a limited number of genetic maps were published, there is no consensus map to date. Alfalfa linkage maps reported so far have variable sizes and marker density depending on the type of mapping population, maker types, and software used. Most studies reported tetraploid alfalfa maps either for 8 linkage groups or for 32 linkage groups for each parent (Julier et al., 2003; Li et al., 2015). An early reported tetraploid alfalfa linkage map covered only 443 cM for seven linkage groups (Brouwer and Osborn, 1999). Julier et al. (2003) constructed alfalfa genetic maps using SSR and AFLP for eight linkage groups containing four homologs, where the parental maps spanned 2649 and 3045 cM (Julier et al., 2003). The authors argued that their alfalfa genetic maps were close to saturation and exhibited high level of collinearity with other maps of alfalfa and *M*. *truncatula* (Julier et al., 2003). However, the genetic maps they constructed were relatively less dense (7–9 cM/marker). Musial et al. (2007) reported alfalfa linkage maps using a backcross (BC) population, where the eight linkage groups spanned 794.1 cM with 3.9 cM/marker (Musial et al., 2007). Li et al. (2015) constructed linkage maps of WISFAL-6 and ABI408 with respective lengths of 898 cM and 845 cM for 8 linkage groups (Li et al., 2015). The linkage map constructed in this study is the second high-density genetic map, published so far, after the alfalfa linkage maps described by Li et al. (2014b) for two alfalfa genotypes DM3 and DM5. Moreover, our linkage maps for both 3010 and CW 1010 parents have almost similar average marker density (~ 1.5 cM/SDA-SNP) to the linkage map of parent DM3 (Li et al., 2014b). Our linkage maps also exhibited high level of synteny with the *M*. *truncatula* genome as in (Julier et al., 2003; Li et al., 2014b). The total length of the 3010 map (2788.5 cM) was slightly higher than the CW 1010 map (2127.55 cM). Such differences in parental linkage maps were also observed in previous alfalfa genetic linkage maps (Julier et al., 2003). The difference might be due to more SDA markers segregating in 3010 parents (5348) compared to (2340) segregating for CW 1010. The higher number of markers in 3010 may be resulted because of higher number of recombinations that lead to a longer linkage map. In *Brassica oleracea*, the recombination frequency in female meiosis is higher than the male, which obviously generates more markers for the female linkage map and therefore a longer map than the male one (Kearsey et al., 1996). However, in alfalfa there are no reports available regarding sex related differences in meiosis frequency. As we obtained a lower number of raw reads for CW 1010 parents than 3010 in either replications, we believe that these differences could result in low number of markers for CW 1010. Furthermore, since GBS is a reduced representation approach, the number and quality of genotype calls may vary between individuals (Gorjanc et al., 2015).

### Mapping and QTL detection

Constructing linkage maps based on single dose markers (1:1) in outcrossing polyploid species and using the maps for linkage analysis of quantitative traits has been a common practice for decades (Wu et al., 1992). The pseudo-testcross strategy uses the heterozygous markers for one parent and double null in other parents for mapping, which further uses inbred backcross configuration in mapping software (Scott, 2004; Wu et al., 2010). This mapping strategy has been successfully used before in tree plants such as *Eucalyptus grandis* and *Eucalyptus urophylla* (Grattapaglia and Sederoff, 1994), *Pinus elliottii* and *P*. *caribaea* (Shepherd et al., 2003), and grass such as orchardgrass (*Dactylis glomerata* L.) (Xie et al., 2010). This mapping strategy possesses however some drawbacks (Pastina et al., 2012). Dominant and additive effects on QTL are confounded and the effects of alleles that were substituted only from other parents are obtainable (Weller, 1992; Boopathi, 2013). Since the parents of the pseudo-testcross population are heterozygous, the marker and QTL alleles may be in different state and linkage phases, which makes the strategy less powerful than the classical QTL analysis in inbred populations (Boopathi, 2013) in addition to mapping only a portion of markers (Semagn et al., 2010). Nevertheless, this strategy is still useful in detecting QTL, displaying the direction and magnitude of QTL effect and the position of QTL in species with complex genome such as polyploids.

In this study, we used the pseudo-testcross strategy with GBS SNPs in alfalfa for QTL mapping of dormancy and WH in alfalfa. A total of 45 QTLs associated with FD and 35 QTLs for WH were mapped on two alfalfa cultivars CW 1010 (male) and 3010 (female) genetic maps. Even though previous studies reported QTL mapping of dormancy in alfalfa, these maps were based on parents relatively close in dormancy and constructed with traditional markers (Zúñiga et al., 2004; Robins et al., 2007; Li et al., 2015). Furthermore, with 11 dormancy classes and 6 classes of WH it is very unlikely to capture the majority of loci underlying these traits in a bi-parental population. The latest alfalfa QTL map reported QTLs for winter injury and FD, but with large QTL intervals (>10 cM) (Li et al., 2015) leading the authors to suggest the need for further research to narrow down QTLs positions. The mapping population was also generated from a dormant × a semi-dormant and winter hardy parents (WISFAL-6 x ABI408) which makes it difficult to capture the alleles underlying non-dormancy and cold susceptibility. In this study, the QTLs were detected within 1-LOD support interval with flanking and peak markers for all QTLs identified (Tables 5–8). Some of the QTLs detected in this study were located in the same genomic locations as previous studies (Li et al., 2015).

Because of the genotype by environment interactions, the QTLs for FD and WH were categorized into stable QTLs that were consistently expressed in more than one environment and potential QTLs that were detected just in an individual environment for one season or only across environments. Considering QTL × E in the analysis enhances the precision of QTL study since the multi-environment QTL test is more powerful in comparison to single-environment analysis (El-Soda et al., 2019). Therefore, to verify the alfalfa WH and FD QTLs detected in our analysis validation studies need to be conducted in other environments using different alfalfa backgrounds.

### Association between FD and WH

Phenotypic and genetic relationship between alfalfa FD and WH has been a matter of debate for a longtime. Earlier studies reported that alfalfa dormancy and WH are phenotypically correlated (*r* = 0.90) and most likely genetically associated leading breeders to use one trait as surrogate to select for the other (Perry et al., 1987; Brummer et al., 2000). Recent studies re-examining this relationship argued for weaker genetic linkages between the traits and suggested that improving one trait by selecting for the other may not be successful (Brummer et al., 2000; Li et al., 2015). Other findings suggested that the relationship between WH and FD in alfalfa depends on the type of germplasm tested (Brummer et al., 2000), implying that the two traits could be manipulated independently (Brummer et al., 2000; Weishaar et al., 2005).

In this study, we observed moderate positive phenotypic correlations between WH and FD in the F1 pseudo-testcross population. The magnitude of correlation however varied with the assessment time of FD. Dormancy measured in fall after clipping on 21 September showed weaker relation to WH than dormancy assessed in winter. Assessing regrowth height for FD in areas with warmer late autumn temperatures may not be reliable and should be delayed to early winter. Nevertheless, for reliable ratings of FD and WH, multi-years data is necessary (Teuber et al., 1998).

The QTL analysis performed in this study revealed that more than 75% (22/28) of the dormancy QTL detected for the 3010 parent did not share genomic regions with winter hardiness QTLs (Tables 5–8). Similarly, for the CW 1010 parent, more than 70% (12/17) dormancy QTLs detected were localized in different genomic regions than winter hardiness QTLs. These results clearly suggest that the two traits are inherited separately and therefore can be genetically manipulated independently in breeding programs (Brummer et al., 2000; Li et al., 2015). The dormancy QTLs (dorm1, dorm2; ndorm1, ndorm4) sharing common genomic regions with winter hardiness QTLs (wh3, ws2, ws5) in the two parents might have been the result of pseudo-linkage resulting from the simultaneous long-term selection for the two traits. It is important to note that a pseudo-testcross population does not provide enough recombination to break apart closely linked loci. Previous QTL mapping work also found few overlapping QTLs for dormancy and winter injury suggesting genetic relation between the traits (Li et al., 2015). Since the two parents used in our study are more phenotypically divergent (FDR 2 for 3010 vs. FDR 10 for CW 1010) in both dormancy and WH than any of the parents used in previous studies, the genetic linkage between the two traits is most likely tighter (Weishaar et al., 2005). The QTLs detected in this study will be valuable addition to the genomic resources for alfalfa breeding programs and to the understanding of the genetic basis of seasonal dormancy and winter hardiness. The segregating non-dormant genotypes with low winter injury generated in this study constitute a valuable germplasm resource to develop winter-hardy non-dormant cultivars.

## Author contributions

LA: contributed to the data collection, analysis, and writing the manuscript. OL: contributed to the winter hardiness data collection. JM: contributed to the phenotypic data collections at the two locations. AM: contributed to the experimental design of the study and writing the manuscript.

### Conflict of interest statement

The authors declare that the research was conducted in the absence of any commercial or financial relationships that could be construed as a potential conflict of interest.

## Footnotes

^1^http://nmsp.cals.cornell.edu/publications/factsheets/factsheet39.pdf, Cornell University. 2008

^2^http://usda.mannlib.cornell.edu/usda/current/CropProdSu/CropProdSu-01-12-2018.pdf

^3^https://www.alfalfa.org/pdf/2017%20NAFA%20Variety%20Leaflet.pdf

^4^https://www.kyazma.nl/docs/JM5Features.pdf

^5^https://www.alfalfa.org/pdf/2014%20NAFA%20Variety%20Leaflet.pdf

^6^https://www.kyazma.nl/docs/JM5Features.pdf

^7^https://www.kyazma.nl/docs/JM5Features.pdf

## References

[B1] Adhikari L. Missaoui A. M. (2017). Nodulation response to molybdenum supplementation in Alfalfa and it's correlation with root and shoot growh in low Ph soil. J. Plant Nutr. 40, 2290–2302. 10.1080/01904167.2016.1264601

[B2] Adhikari L. Mohseni-Moghadam M. Missaoui A. (2018). Allelopathic effects of cereal rye on weed suppression and forage yield in Alfalfa. Am. J. Plant Sci. 9, 685–700. 10.4236/ajps.2018.94054

[B3] Adhikari L. Razar R. M. Paudel D. Ding R. Missaoui A. M. (2017). Insights into seasonal dormancy of perennial herbaceous forages. Am. J. Plant Sci. 8, 2650 10.4236/ajps.2017.811179

[B4] Bolger A. Giorgi F. (2014). Trimmomatic: A Flexible Read Trimming Tool for illumina NGS Data. Available online at: http://www.usadellab.org/cms/index.php.

[B5] Boopathi N. M. (2013). Genetic Mapping and Marker Assisted Selection: Basics, Practice and Benefits. New Delhi: Springer India.

[B6] Boudhrioua C. Bastien M. Légaré G. Pomerleau S. St-Cyr J. Boyle B.. (2017). Genotyping-by-sequencing in potato, in The Potato Genome, eds Kumar Chakrabarti S. Xie C. Kumar Tiwari J. (Cham: Springer International Publishing), 283–296.

[B7] Brouwer D. J. Osborn T. C. (1999). A molecular marker linkage map of tetraploid alfalfa (*Medicago sativa L*.). Theor. Appl. Genet. 99, 1194–1200. 10.1007/s001220051324

[B8] Brouwer D. J. Duke S. H. Osborn T. C. (2000). Mapping genetic factors associated with winter hardiness, fall growth, and freezing injury in autotetraploid alfalfa. Crop Sci. 40, 1387–1396. 10.2135/cropsci2000.4051387x

[B9] Brummer E. C. Shah M. M. Luth D. (2000). Reexamining the relationship between fall dormancy and winter hardiness in alfalfa. Crop Sci. 40, 971–977. 10.2135/cropsci2000.404971x

[B10] Castonguay Y. Bertrand A. Michaud R. Laberge S. (2011). Cold-induced biochemical and molecular changes in alfalfa populations selectively improved for freezing tolerance. Crop Sci. 51, 2132–2144. 10.2135/cropsci2011.02.0060

[B11] Cunningham S. M. Volenec J. J. Teuber L. R. (1998). Plant survival and root and bud composition of alfalfa populations selected for contrasting fall dormancy. Crop Sci. 38, 962–969. 10.2135/cropsci1998.0011183X003800040014x

[B12] da Silva W. L. Ingram J. Hackett C. A. Coombs J. J. Douches D. Bryan G. J.. (2017). Mapping loci that control tuber and foliar symptoms caused by PVY in autotetraploid Potato (*Solanum tuberosum L*.). G3 (Bethesda) 7, 3587–3595. 10.1534/g3.117.300264 28903982 PMC5675608

[B13] Dhanaraj A. L. Alkharouf N. W. Beard H. S. Chouikha I. B. Matthews B. F. Wei H.. (2007). Major differences observed in transcript profiles of blueberry during cold acclimation under field and cold room conditions. Planta 225, 735–751. 10.1007/s00425-006-0382-1 16953429

[B14] Doyle J. Doyle J. (1987). Genomic plant DNA preparation from fresh tissue-CTAB method. Phytochem. Bull 19, 11–15.

[B15] Duke JA (1981). Handbook of Legumes of World Economic Importance. New York, NY: Plenum Press.

[B16] Elshire R. J. Glaubitz J. C. Sun Q. Poland J. A. Kawamoto K. Buckler E. S.. (2011). A Robust, Simple Genotyping-by-Sequencing (GBS) Approach for High Diversity Species. PLoS ONE 6:e19379. 10.1371/journal.pone.0019379 21573248 PMC3087801

[B17] El-Soda M. Malosetti M. Zwaan B. J. Koornneef M. Aarts G. M. (2019). Genotype × environment interaction QTL mapping in plants: lessons from Arabidopsis. Trends Plant Sci. 19, 390–398. 10.1016/j.tplants.2014.01.001 24491827

[B18] Eujayl I. Sledge M. K. Wang L. May G. D. Chekhovskiy K. Zwonitzer J. C.. (2004). Medicago truncatula EST-SSRs reveal cross-species genetic markers for *Medicago* spp. Theor. Appl. Genet. 108, 414–422. 10.1007/s00122-003-1450-6 13679975

[B19] Gar O. Sargent D. J. C.-Tsai J. Pleban T. Shalev G. Zamir D.. (2011). An autotetraploid linkage map of Rose (*Rosa hybrida*) validated using the Strawberry (*Fragaria vesca*) genome sequence. PLoS ONE 6:e20463. 10.1371/journal.pone.0020463 21647382 PMC3103584

[B20] Gorjanc G. Cleveland M. A. Houston R. D. Hickey J. M. (2015). Potential of genotyping-by-sequencing for genomic selection in livestock populations. Genet. Select. Evolut. 47, 12. 10.1186/s12711-015-0102-z 25887531 PMC4344748

[B21] Grattapaglia D. Sederoff R. (1994). Genetic linkage maps of Eucalyptus grandis and Eucalyptus urophylla using a pseudo-testcross: mapping strategy and RAPD markers. Genetics 137, 1121–1137. 7982566 10.1093/genetics/137.4.1121 PMC1206059

[B22] Gusta L. V. Wisniewski M. (2013). Understanding plant cold hardiness: an opinion. Physiol. Plant. 147, 4–14. 10.1111/j.1399-3054.2012.01611.x 22409670

[B23] Hackett C. A. Boskamp B. Vogogias A. Preedy K. F. Milne I. (2017). TetraploidSNPMap: software for linkage analysis and QTL mapping in autotetraploid populations using SNP dosage data. J. Heredity 108, 438–442. 10.1093/jhered/esx022

[B24] Hackett C. A. Milne I. Bradshaw J. E. Luo Z. (2007). TetraploidMap for Windows: linkage map construction and QTL mapping in autotetraploid species. J. Hered. 98, 727–729. 10.1093/jhered/esm086 17965198

[B25] Haggard J. E. Johnson E. B. St. Clair D. A. (2015). Multiple QTL for horticultural traits and quantitative resistance to *Phytophthora infestans* linked on *Solanum habrochaites* chromosome 11. G3 (Bethesda) 5, 219–233. 10.1534/g3.114.014654 25504736 PMC4321030

[B26] Ibáñez C. Kozarewa I. Johansson M. Ögren E. Rohde A. Eriksson M. E. (2010). Circadian clock components regulate entry and affect exit of seasonal dormancy as well as winter hardiness in populus trees. Plant Physiol. 153, 1823–1833. 10.1104/pp.110.158220 20530613 PMC2923903

[B27] Julier B. Flajoulot S. Barre P. Cardinet G. Santoni S. Huguet T.. (2003). Construction of two genetic linkage maps in cultivated tetraploid alfalfa (*Medicago sativa*) using microsatellite and AFLP markers. BMC Plant Biol. 3:9. 10.1186/1471-2229-3-9 14683527 PMC324403

[B28] Kearsey M. J. Ramsay L. D. Jennings D. E. Lydiate D. J. Bohuon E. J. Marshall D. F.. (1996). Higher recombination frequencies in female compared to male meisoses in Brassica oleracea. Theor. Appl. Genet. 92, 363–367. 10.1007/BF00223680 24166258

[B29] Li H. Handsaker B. Wysoker A. Fennell T. Ruan J. Homer N.. (2009). The sequence alignment/map format and SAMtools. Bioinformatics 25, 2078–2079. 10.1093/bioinformatics/btp352 19505943 PMC2723002

[B30] Li X. Alarcón-Zú-iga B. Kang J. Nadeem Tahir M. H. Jiang Q. Wei Y. (2015). Mapping fall dormancy and winter injury in tetraploid alfalfa. Crop Sci. 55, 1995–2011. 10.2135/cropsci2014.12.0834

[B31] Li X. Brummer E. C. (2012). Applied genetics and genomics in alfalfa breeding. Agronomy 2:40 10.3390/agronomy2010040

[B32] Li X. Han Y. Wei Y. Acharya A. Farmer A. D. Ho J.. (2014a). Development of an Alfalfa SNP array and its use to evaluate patterns of population structure and linkage disequilibrium. PLoS ONE 9:e84329. 10.1371/journal.pone.0084329 24416217 PMC3887001

[B33] Li X. Wei Y. Acharya A. Jiang Q. Kang J. Brummer E. C. (2014b). A saturated genetic linkage map of autotetraploid alfalfa (*Medicago sativa L*.) developed using genotyping-by-sequencing is highly syntenous with the *Medicago truncatula* genome. G3 (Bethesda) 4, 1971–1979. 10.1534/g3.114.012245 25147192 PMC4199703

[B34] Li X. Wei Y. Moore K. J. Michaud R. Viands D. R. Hansen J. L. (2011). Association mapping of biomass yield and stem composition in a tetraploid alfalfa breeding population. Plant Genome 4, 24–35. 10.3835/plantgenome2010.09.0022 33228301

[B35] Lu F. Lipka A. E. Glaubitz J. Elshire R. Cherney J. H. Casler M. D.. (2013). Switchgrass genomic diversity, ploidy, and evolution: novel insights from a network-based SNP discovery protocol. PLoS Genet. 9:e1003215. 10.1371/journal.pgen.1003215 23349638 PMC3547862

[B36] McCaslin M. Woodward T. Undersander D. (2003). Winter Survival, Standard Tests to Characterize Alfalfa Cultivars. Beltsville, MD: North America Alfalfa Improvement Conference Available online at: http://www.naaic.org/stdtests/wintersurvivalnew.htm (Accessed May 20, 2013).

[B37] McKenzie J. S. Paquin R. Duke S. H. (1988). Cold and Heat Tolerance, in Alfalfa and Alfalfa Improvement, American Society of Agronomy, Crop Science Society of America, Soil Science Society of America, eds Hanson A. A. Barnes D. K. Hill R. R. (Madison, WI), 259–302.

[B38] Melo A. T. O. Bartaula R. Hale I. (2016). GBS-SNP-CROP: a reference-optional pipeline for SNP discovery and plant germplasm characterization using variable length, paired-end genotyping-by-sequencing data. BMC Bioinformatics 17:29. 10.1186/s12859-016-0879-y 26754002 PMC4709900

[B39] Moriguchi Y. Ujino-Ihara T. Uchiyama K. Futamura N. Saito M. Ueno S.. (2012). The construction of a high-density linkage map for identifying SNP markers that are tightly linked to a nuclear-recessive major gene for male sterility in *Cryptomeria japonica* Don, D. BMC Genomics 13:95. 10.1186/1471-2164-13-95 22424262 PMC3386010

[B40] Musial J. M. Mackie J. M. Armour D. J. Phan H. T. Ellwood S. E. Aitken K. S.. (2007). Identification of QTL for resistance and susceptibility to Stagonospora meliloti in autotetraploid lucerne. Theor. Appl. Genet. 114, 1427–1435. 10.1007/s00122-007-0528-y 17356865

[B41] Olsen J. E. (2010). Light and temperature sensing and signaling in induction of bud dormancy in woody plants. Plant Mol. Biol. 73, 37–47. 10.1007/s11103-010-9620-9 20213333

[B42] Pastina M. Malosetti M. Gazaffi R. Mollinari M. Margarido G. Oliveira K.. (2012). A mixed model QTL analysis for sugarcane multiple-harvest-location trial data. Theor. Appl. Genet. 124, 835–849. 10.1007/s00122-011-1748-8 22159754 PMC3284670

[B43] Perry M. C. McIntosh M. S. Wiebold W. J. Welterlen M. (1987). Genetic analysis of cold hardiness and dormancy in alfalfa. Genome 29, 144–149. 10.1139/g87-024

[B44] Robins J. Luth D. Campbell T. Bauchan G. He C. Viands D. (2007). Genetic mapping of biomass production in tetraploid alfalfa. Crop Sci. 47, 1–10. 10.2135/cropsci2005.11.0401

[B45] SAS Institute Inc (2004). Cary, NC: SAS Institute Inc.

[B46] Santhosh K. (1989). http://bio-bwa.sourceforge.net/.

[B47] Scott L. J. (2004). Implications of evolutionary history and population structure for the analysis of quantitative trait loci in the ancient conifer Araucaria cunninghamii. PhD thesis, Southern Cross University, Lismore, NSW.

[B48] Semagn K. Bjørnstad Å. Xu Y. (2010). The genetic dissection of quantitative traits in crops. Electr. J. Biotechnol. 13, 16–17. 10.2225/vol13-issue5-fulltext-14

[B49] Shepherd M. Cross M. Dieters M. J. Henry R. (2003). Genetic maps for *Pinus elliottii var*. elliottii and P. caribaea var. hondurensis using AFLP and microsatellite markers. Theor. Appl. Genet. 106, 1409–1419. 10.1007/s00122-002-1185-9 12750783

[B50] Shu Y. Li W. Zhao J. Zhang S. Xu H. Liu Y.. (2017). Transcriptome sequencing analysis of alfalfa reveals CBF genes potentially playing important roles in response to freezing stress. Genet. Mol. Biol. 40, 824–833. 10.1590/1678-4685-gmb-2017-0053 29111565 PMC5738619

[B51] Stout D. G. Hall J. W. (1989). Fall growth and winter survival of alfalfa in interior British Columbia. Can. J. Plant Sci. 69, 491–499. 10.4141/cjps89-060

[B52] Tanino K. K. Kalcsits L. Silim S. Kendall E. Gray G. R. (2010). Temperature-driven plasticity in growth cessation and dormancy development in deciduous woody plants: a working hypothesis suggesting how molecular and cellular function is affected by temperature during dormancy induction. Plant Mol. Biol. 73, 49–65. 10.1007/s11103-010-9610-y 20191309

[B53] Teuber L. Taggard K. Gibbs L. McCaslin M. Peterson M. Barnes D. (1998). Fall dormancy, Standard tests to characterize alfalfa cultivars, in 36th international Conference the North American Alfalfa Improvement (Bozeman, MT), 2–6.

[B54] Voorrips R. E. (2002). MapChart: software for the graphical presentation of linkage maps and QTLs. J. Hered. 93, 77–78. 10.1093/jhered/93.1.77 12011185

[B55] Wan S. M. Liu H. Zhao B. W. Nie C. H. Wang W. M.. (2017). Construction of a high-density linkage map and fine mapping of QTLs for growth and gonad related traits in blunt snout bream. Sci. Rep. 7:46509. 10.1038/srep46509 28422147 PMC5395971

[B56] Weishaar M. A. Brummer E. C. Volenec J. J. Moore K. J. Cunningham S. (2005). Improving Winter Hardiness in Nondormant Alfalfa Germplasm This journal paper of the Iowa Agric. Home Econ. Exp. Stn., Ames, IA, Project No. 6631 and 6525, was supported by the Hatch Act and State of Iowa funds. Crop Sci. 45, 60–65. 10.2135/cropsci2005.0060

[B57] Weller J. I. (1992). Statistical methodologies for mapping and analysis of quantitative trait loci, in Plant Genomes: Methods for Genetic and Physical Mapping. eds Beckmann J. S. Osborn T. C. (Dordrecht: Springer), 181–207.

[B58] Wu K. Burnquist W. Sorrells M. Tew T. Moore P. Tanksley S. (1992). The detection and estimation of linkage in polyploids using single-dose restriction fragments. Theor. Appl. Genet. 83, 294–300. 10.1007/BF00224274 24202510

[B59] Wu S. Yang J. Huang Y. Li Y. Yin T. Wullschleger S. D.. (2010). An improved approach for mapping quantitative trait loci in a pseudo-testcross: revisiting a poplar mapping study. Bioinform. Biol. Insights 4, 1–8. 10.4137/BBI.S4153 20213011 PMC2832300

[B60] Xie W. Zhang X. Cai H. Huang L. Peng Y. Ma X. (2010). Genetic maps of SSR and SRAP markers in diploid orchardgrass (*Dactylis glomerata* L.) using the pseudo-testcross strategy. Genome 54, 212–221. 10.1139/G10-111 21423284

[B61] Xiong Y. Fei S.-Z. Arora R. Brummer E. C. Barker R. E. Jung G. (2007). Identification of quantitative trait loci controlling winter hardiness in an annual × perennial ryegrass interspecific hybrid population. Mol. Breed. 19, 125–136. 10.1007/s11032-006-9050-1

[B62] Zhang S. Shi Y. Cheng N. Du H. Fan W. Wang C. (2015). *De novo* characterization of fall dormant and nondormant Alfalfa (*Medicago sativa L*.) leaf transcriptome and identification of candidate genes related to fall dormancy. PLoS ONE 10:e0122170. 10.1371/journal.pone.0122170 25799491 PMC4370819

[B63] Zúñiga B. A. Scott P. Moore K. Luth D. Brummer E. (2004). Quantitative Trait Locus Mapping of Winter Hardiness Metabolites in Autotetraploid Alfalfa (*M. sativa*). Molecular Breeding of Forage and Turf. Developments in Plant Breeding, Vol. 11, eds Hopkins A. Wang Z.Y. Mian R. Sledge M. Barker R. E. (Dordrecht: Springer), 97–104.

