# Supplementary material for: Dissecting Key Adaptation Traits in the Polyploid Perennial Medicago sativa Using GBS-SNP Mapping
Source: Front Plant Sci. 2018 Jul 4;9:934. doi: 10.3389/fpls.2018.00934 (PMC6039623; doi:10.3389/fpls.2018.00934)
Supplement: Supplementary file 1 [file Data_Sheet_1.DOCX]

Supplementary figure S1

Figure S1: Thirty-two linkage groups for the maternal parent 3010 map. The positions of SNPs were given in Kosambi centimorgan (cM). Four haplotype maps (A, B, C, D) were grouped per chromosome based on the positions of SNPs obtained from BLAST analysis using *M*. *truncatula* reference genome.
