# Supplementary material for: Dissecting Key Adaptation Traits in the Polyploid Perennial Medicago sativa Using GBS-SNP Mapping
Source: Front Plant Sci. 2018 Jul 4;9:934. doi: 10.3389/fpls.2018.00934 (PMC6039623; doi:10.3389/fpls.2018.00934)
Supplement: Supplementary file 2 [file Data_Sheet_2.DOCX]

Supplementary figure S2

Figure S2: Thirty-two linkage groups for the paternal parent CW1010 map. The positions of SNPs were given in Kosambi centimorgan (cM). Four haplotype maps (A, B, C, D) were grouped per chromosome based on the positions of SNPs obtained from BLAST analysis using *M*. *truncatula* reference genome.
